# Topology optimization of the flat steel shear wall based on the volume constraint and strain energy assumptions under the seismic loading conditions

**DOI:** 10.1038/s41598-024-61204-1

**Published:** 2024-05-06

**Authors:** Xi Chen, Gongxing Yan, Hasan Hosseinzadeh

**Affiliations:** 1 School of Architecture and Engineering, Chongqing Industry & Trade Polytechnic, Chongqing, 408300 China; 2 School of Intelligent Construction, Luzhou Vocational and Technical College, Luzhou, 646000 Sichuan China; 3 Luzhou Key Laboratory of Intelligent Construction and Low-Carbon Technology, Luzhou, 646000 Sichuan China; 4 Department of Mathematics, Ardabil Branch, Islamic Azad University, Ardabil, Iran; 5 College of Technical Engineering, The Islamic University, Najaf, Iraq

**Keywords:** Steel shear wall, Infill plate, Topology optimization, Finite element analysis, Cyclic load conditions, Engineering, Materials science

## Abstract

In structural engineering systems, shear walls are two-dimensional vertical elements designed to endure lateral forces acting in-plane, most frequently seismic and wind loads. Shear walls come in a variety of materials and are typically found in high-rise structures. Because steel shear walls are lighter, more ductile, and stronger than other concrete shear walls, they are advised for usage in steel constructions. It is important to remember that the steel shear wall has an infill plate, which can be produced in a variety of forms. The critical zones in flat steel shear walls are the joints and corners where the infill plate and frame meet. The flat infill plate can be modified to improve the strength and weight performance of the steel shear walls. One of these procedures is Topology Optimization (TO) and this method can reduce the weight and also, increase the strength against the cyclic loading sequences. In the current research paper, the TO of the infill steel plate was considered based on the two methods of volume constraint and maximization of strain energy. Four different volumes (i.e., 60%, 70%, 80%, and 90%) were assumed for the mentioned element in the steel shear wall. The obtained results revealed that the topology configuration of CCSSW with 90% volume constraint presented the highest seismic loading performance. The cumulated energy for this type of SSW was around 700 kJ while it was around 600 kJ for other topology optimization configurations.

## Introduction

Cyclic loading is the process of applying repeated or variable strains, stresses, or stress intensities to various locations on structural components^1^. The term “fatigue degradation” refers to potential degradation and localized damage^2^. Shear walls are one of the responsibilities that are taken into consideration throughout the design and manufacturing process of structures in order to reduce damages^3^. A shear wall is a structural support element that can withstand shear stresses, including those caused by severe winds and seismic activity^4^. In the field of civil engineering, shear force refers to the forces that apply perpendicularly against the structural components of a building, causing the structure to twist and bend^5^. Shear walls make structures stable and prevent them from tilting or collapsing by transferring lateral loads to the base^6^. Shear walls all serve the same purpose, but they must follow specific construction guidelines and can vary based on several building-related factors, such as the material they are made of (such as concrete^7^, steel^8^, or wood^9^), their thickness^10^, length^11^, and placement^12^. The purpose of load-bearing and shear walls is to transfer the pressure and tension associated with a load from its source to the ground foundation. Load-bearing walls, on the other hand, absorb vertical loads and hold a building up, whereas shear walls resist horizontal forces and prevent a building from tumbling sideways^13^. Shear walls can be made from a variety of materials, but the most common ones are plywood, steel, and concrete. Reinforced concrete is used in the building of shear walls to increase their stiffness and lateral strength. These concrete walls are most frequently used in medium to high-rise buildings^14^. Plywood shear walls, which are designed to withstand wind loads from hurricanes, tornadoes, and severe winds, are meant to be able to tolerate continuous lateral strains^15^. Steel plate shear walls are constructed using steel and infill plates. They are most commonly used in tall structures for seismic protection because they are made with boundary characteristics that can bear seismic stresses during events like earthquakes^16^. According to the points raised, steel shear walls are typically employed in steel constructions because they are lighter, more ductile, and stronger than other kinds of shear walls^17^. As previously mentioned, the steel shear walls consist of several components, such as columns, beams, and an infill plate. A key component in regulating the steel shear wall’s seismic response is the infill plate^18^.

One of the key points in the field of strengthening the shear walls, is topology optimization. As a form optimization technique, topology optimization makes use of mathematical models to optimize material arrangement for a given set of loads, circumstances, and constraints inside a user-defined space^19^. By considering the topology optimization on the steel shear walls, the weight and strength can be reduced and improved, respectively. Therefore, topology optimization can be useful from a cyclic performance point of view.

In order to gain a better understanding of the seismic performances of shear walls, several researchers have recently attempted to investigate various techniques that may be effective against the seismic sequences. Some of the published articles about the aforementioned issues are included in the paragraph that follows.

The findings of Khan and Srivastava’s^20^ study on the inelastic behavior of steel plate shear walls under various opening conditions were published, and they showed that the opening systems had a major impact. Lastly, they offered novel models for the degradations of stiffness and shear strength. The greater length of the infill plate led to a better energy dissipation capability, and Mansouri et al.^21^ verified that the higher amounts of applied energy were dissipated through the infill plate. Using 57 computer models, Paslar et al.^22^ investigated how the border elements and the infill plate of a steel plate shear wall are connected. Additionally, they used the experimental models to confirm the initial ones. In actuality, it was determined that a significant component was the way the infill plate and frame were connected. The obtained results showed that in comparison to the column-only connected joint, the beam-only connected joint decreased the shear force resistive in the shear wall. The primary objective of the research has been to come up with novel approaches to enhance the steel plate shear walls’ ductility, energy absorption, and other properties. For example, Lu et al.^23^ suggested that the seismic load-resisting system in the steel structures be self-centering steel plate shear walls with slits. Finite element analysis proved the indicated shear walls’ capabilities. When compared to traditional shear walls, Azandariani et al.’s^24^ study showed the efficacy of the partial plate-column connection in steel plate shear walls.

Professional software is now made specifically for topology optimization thanks to the development of optimization techniques. Numerous research that optimizes the topology of structures has recently been reported. Topology optimization was utilized by Bagherinejad and Haghollahi^25^ to determine the optimal form of perforated steel plate shear wall in the moment frames. They used the maximizing of reaction forces as the objective function and carried out the optimization. Daryan et al.^26^ proposed an effective approach for the best design of frames with steel plate shear walls by utilizing bat optimization and modified dolphin echolocation techniques.

According to the above literature, using topology optimization to find the optimum shape of the infill plates of the steel shear walls can be useful in the presence of cyclic loading conditions. Thus, in this research article, three different connection types between the infill plate and the border elements (i.e., fully-connected, beam-connected, and column-connected) together with the two well-known methods of volume and strain energy were assumed for the analyses through the use of finite element analyses. Besides, four various volume constraints of 60%, 70%, 80%, and 90% were remarked for the infill plates of the steel shear walls. It is noteworthy that the selected assumption is considered for the first time and it can be stated that this paper considered comprehensive assumptions. Then, finite element analysis was implemented to figure out the optimum infill plate shapes and some critical characteristics of the steel shear walls including stress and strain distributions, hysteresis curves, strength, cumulated energy, and plastic dissipation energy.

## Methodology

As stated earlier, the current research paper used the topology optimization concept in the steel shear walls to obtain the optimum shape of the infill plate of the steel shear wall by utilizing finite element simulations. Thus, first, this concept is briefly described and then the implemented experimental data together with the studied steel shear walls in the current research paper are stated.

### Topology optimization (TO) methodology

The theory of TO is described here, along with the objective function, constraints, solution method, and general formulation. It should be noted that the Topology Optimization (TO) concepts are stated in this subsection to provide better clarifications about the TO’s main formulations for the readers. However, TO procedures were carried out employing the Tosca plugin within Abaqus CAE. The volume constraints and strain energy minimization are the TO’s objective function in this research. The energy bilinear form serves as the foundation for the formulation of TO employing gradient techniques such as sensitivity analysis^27^. Furthermore, it is feasible to perform a nonlinear FEA in a TO using the strain energy. A scalar parameter called strain energy (*Π*) represents the stored energy brought about by structural deformations. The energy is equivalent to the product of stress (*σ*) and strain (*ε*) on the element’s volume (*V*). The sum of the strain energies in each element of a structure equals the total strain energy of the structure (see Eq. (1)).

1 $$ \Pi = \frac{1}{2}\int {\int {\int {\left\{ \varepsilon \right\}^{T} \left\{ \sigma \right\}dV = \frac{1}{2}\sum\limits_{e = 1}^{N} {u_{e}^{T} k_{e} u_{e} } } } } $$

where the element displacement vector (*u*_*e*_) and the stiffness matrix (*k*_*e*_), respectively, are shown. When a displacement pattern is applied to the structure as the loading, the strain energy should be maximized to maximize the structural stiffness^27^. 60%, 70%, 80%, and 90% of the primary infill plate were the defined restrictions for the final volume of the optimized plate. To do the TO, there are three fundamental steps. Based on the findings of the finite element analysis, the first step is to determine the effective elements of the objective function. In the second stage, a penalization mechanism must be used to enhance and reduce, respectively, the density and stiffness of the effective and ineffective elements. All elements’ density variables need to be adjusted in the third phase. Iteratively carrying out these actions is necessary until the stop criteria and the constraint are satisfied. Two well-known gradient methods are sensitivity analysis and optimality criteria. Here, the effective elements are found using sensitivity analysis, the density of the elements is changed using Simple Isotropic Material with Penalization (SIMP), and the design variables are updated using the Method of Moving Asymptotes (MMA). The SIMP-based TO formulation can be expressed as follows:

2 $$ \max :\psi_{(\rho )} = \sum\limits_{e = 1}^{N} {\left( {\rho_{e} } \right)}^{P} u_{e}^{T} k_{e} u_{e} $$

3 $$ Subject \, to:\left\{ {\begin{array}{*{20}l} {\frac{{V_{(x)} }}{{V_{o} }}} \hfill \\ {KU = F} \hfill \\ {0 < \rho_{\min } \le \rho_{e} \le 1} \hfill \\ \end{array} } \right. $$

where *F* is the prescribed volume fraction (*f* = 0.50), *Ψ* is the objective function, *ρ*_*e*_ is the density of the elements (the design variable), *p* is the penalization power (*p* = 3), *V*_*(x)*_ and *V*_*o*_ are the material volume and design domain volume, respectively, *K* is the global stiffness matrix, *ρ*_*min*_ is the minimum relative density (*ρ*_*min*_ = 0.001), and *N* is the number of elements used to discretize the design domain. According to^27^, *p* = 3 is an appropriate value based on the numerical results of completed experiments. The following could be used to express how sensitive strain energy (the objective function) is to element density (the design variable):

4 $$ \frac{\partial \psi }{{\partial \rho_{e} }} = \frac{\partial }{{\partial \rho_{e} }}\left( {U^{T} KU} \right) = 2U^{T} K\frac{\partial U}{{\partial \rho_{e} }} + U^{T} \frac{\partial K}{{\partial \rho_{e} }}U $$

Taking into account the generic static equation (*KU* = *F*):

5 $$ \frac{\partial }{{\partial \rho_{e} }}\left( {KU - F} \right) = \frac{\partial K}{{\partial \rho_{e} }}U + \frac{\partial U}{{\partial \rho_{e} }}K = 0 \to \frac{\partial U}{{\partial \rho_{e} }}K = - \frac{\partial K}{{\partial \rho_{e} }}U $$

Using Eq. (5) in place with Eq. (4):

6 $$ \frac{\partial \psi }{{\partial \rho_{e} }} = - U^{T} \frac{\partial K}{{\partial \rho_{e} }}U $$

The following is one possible way to write Eq. (6) using the SIMP approach and Eq. (2):

7 $$ \frac{\partial \psi }{{\partial \rho_{e} }} = - p\rho_{e}^{p - 1} \sum {u_{e}^{T} k_{e} u_{e} } $$

The solution method and mechanism for updating the element density in each iteration is the Method of Moving Asymptotes (MMA). By using Eq. (8), MMA approximates a function (*Ψ*) of n real variables *x* = (*x*_*1*_. … .*x*_*n*_) around a given iteration point *x*^*0*^.

8 $$ \begin{aligned} & \psi \approx \psi (x^{0} ) + \sum\limits_{i = 1}^{n} {\left( {\frac{{r_{i} }}{{U_{i} - x_{i} }} + \frac{{s_{i} }}{{x_{i} - L_{i} }}} \right)} \\ & Subject\; \, to: \\ & \left\{ {\begin{array}{*{20}l} {if\;\frac{\partial \psi }{{\partial x_{i} }}(x^{0} ) > 0\;{\text{ then}}\; \, r_{i} = (U_{i} - x_{i}^{0} )^{2} \frac{\partial \psi }{{\partial x_{i} }}(x^{0} )\;{\text{ and }}\;s_{i} = 0} \hfill \\ {if\;\frac{\partial \psi }{{\partial x_{i} }}(x^{0} ) < 0{\text{ then }}r_{i} = 0\;{\text{ and}}\; \, s_{i} = - (x_{i}^{0} - L_{i} )^{2} \frac{\partial \psi }{{\partial x_{i} }}(x^{0} )} \hfill \\ \end{array} } \right\} \\ \end{aligned} $$

where the top and lower vertical asymptotes for the approximations of *Ψ* are denoted by *U*_*i*_ and *L*_*i*_, respectively. The sensitivity of strain energy is shown to be negative for all element densities by Eq. (7). Thus, following iteration *j*, the MMA formulation (Eq. (8)) could be expressed as Eq. 9^27^.

9 $$ \begin{aligned} & Max: \, \left\{ {\psi_{{(\rho^{i} )}} - \sum\limits_{e = 1}^{N} {\left( {\frac{{(\rho_{e}^{j} - L_{e} )^{2} }}{{\rho_{e} - L_{e} }} \times \frac{\partial \psi }{{\partial \rho_{e} }}(\rho^{j} )} \right)} } \right\} \\ & Subject\; \, to: \, \frac{\partial \psi }{{\partial \rho_{e} }} = - p\left( {\rho_{e} } \right)^{p - 1} u_{e}^{T} k_{e} u_{e} \, \;{\text{and }}\;\sum\limits_{i = 1}^{N} {\rho_{e} \upsilon_{e} \le fV_{0} } \\ \end{aligned} $$

where *υ*_*e*_ is an element’s volume and *L*_*e*_ is the MMA method’s lower bound that could be modified in each iteration. The number of iterations that equals 100 is the initial stop condition. Additionally, two stop conditions are offered in the TO in order to avoid performing additional iterations. According to Eq. (10), these two stop criteria comprise the average changes of element density (*l*_*2*_) and the rate changes of the objective function (*l*_*1*_).

10 $$ \frac{{\left| {\psi_{n + 1} - \psi_{n} } \right|}}{{\left| {\psi_{n + 1} } \right|}} \le l_{1} \, \;and\; \, \frac{{\left| {\rho_{n + 1} - \rho_{n} } \right|}}{N} \le l_{2} $$

**Figure 1:**
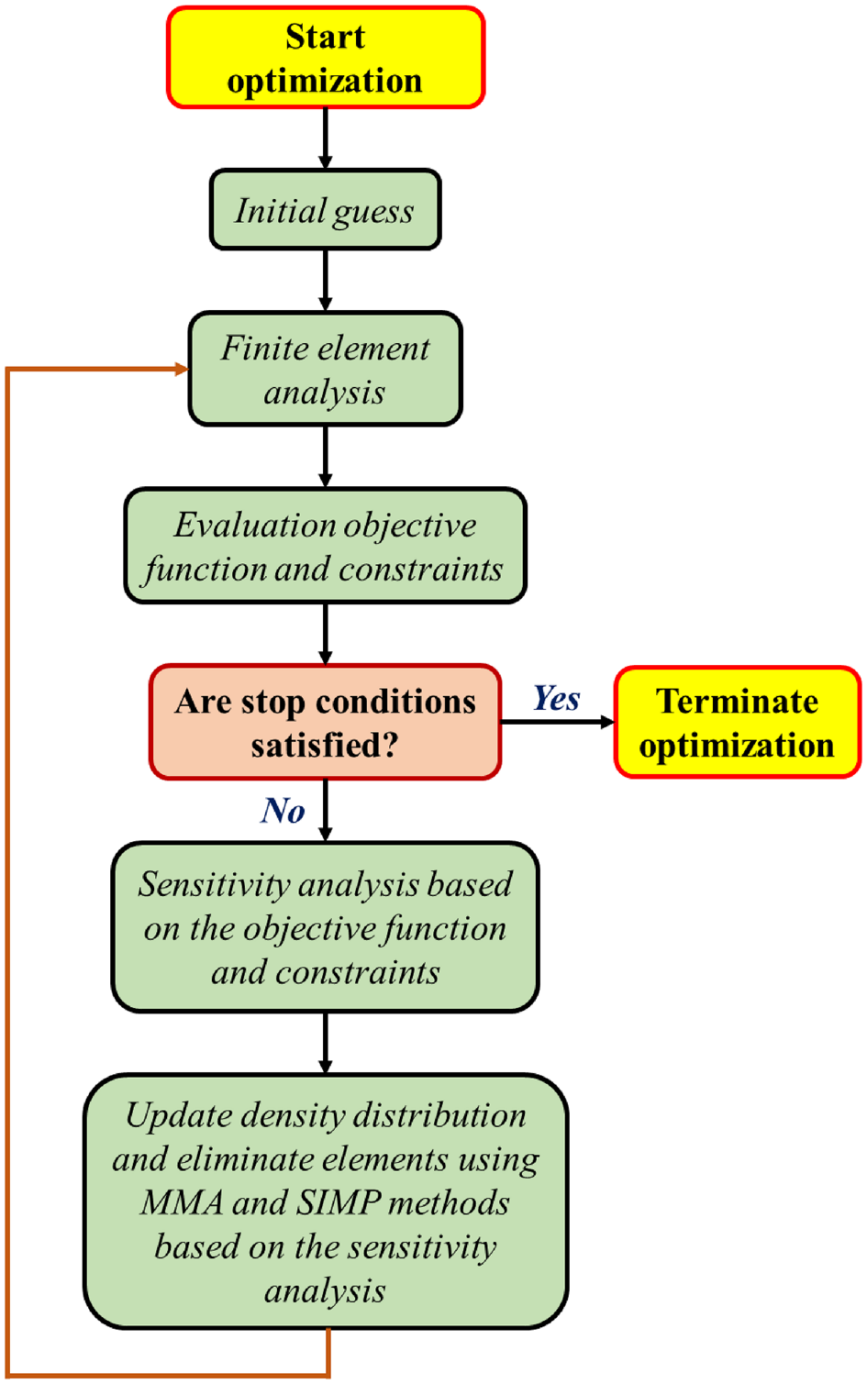
Topology optimization flowchart.

Figure 1 presents a flowchart of the TO processes based on the SIMP, MMA, and sensitivity analysis approaches.

### Experimental data

**Figure 2:**
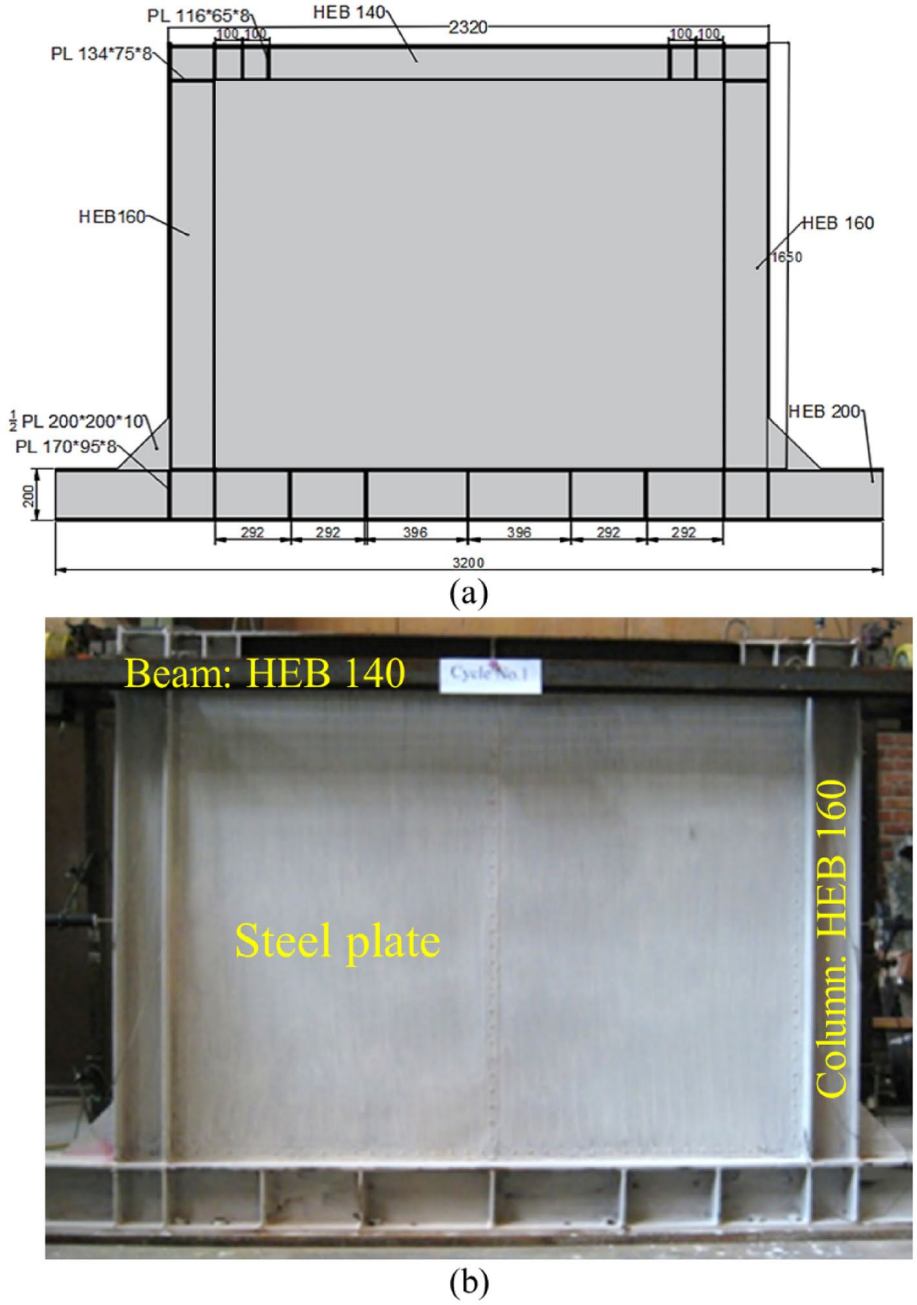
(**a**) schematic illustration and (**b**) real tested specimen of the fabricated flat steel shear wall^28^.

Here, the reported results by Emami et al.^28^ were considered as the main input data for the analyses. They tested three different types of shear walls; flat Steel Shear Walls (SSWs), vertically Corrugated Steel Shear Walls (CSSWs), and horizontal ones. The results revealed that the corrugated shear walls could present higher cyclic performance compared to the flat SSW. But from an economic issues point of view, the flat SSW was a good choice. The schematic representation and the real test specimen of the flat SSW are exhibited in Fig. 2.

In Fig. 2a, some technical codes are reported which relate to the technical information about the construction of the flat SSW. These technical data are gathered in Table 1.

**Table 1:** Technical data of the flat SSW reported by Emami et al.^28^.

Beam	Column	Plate thickness	L/t	h_s_/t	Type panel
HE-B140	HE-B160	1.25	1600	1200	Flat

The main concepts and reported results of the current research paper rely on the basis of the flat SSW. The objectives of this research study are noted in the next subsection.

### Studied cases and purposes

As stated earlier, the steel shear walls play a vital role in controlling the damping the seismic loading sequences in the steel structures. It should be noted that in the steel shear walls, the infill plate is the major effective element among the other elements naming beam and column. Different shapes are recommended for the infill plate based on the literature review^29^ but still, more endeavors are needed to improve the infill plate’s performance against the cyclic loading conditions. For instance, some researchers proposed corrugated steel shear walls with different corrugation shapes^30^. Here, the current research paper used Topology Optimization (TO) to obtain the optimum shape of the infill steel plate. To optimize the infill plate according to the TO theories, four different volumes of 60%, 70%, 80%, and 90% were stipulated. For clarification, the 60% meant that the optimized infill plate had 60% volume of the initial infill plate. Another constraint that was considered was the minimization of the strain energy in the infill plates of the SSW during the cyclic loading conditions. This assumption resulted in lowering the amounts of the stresses in the concentration zones.

**Figure 3:**
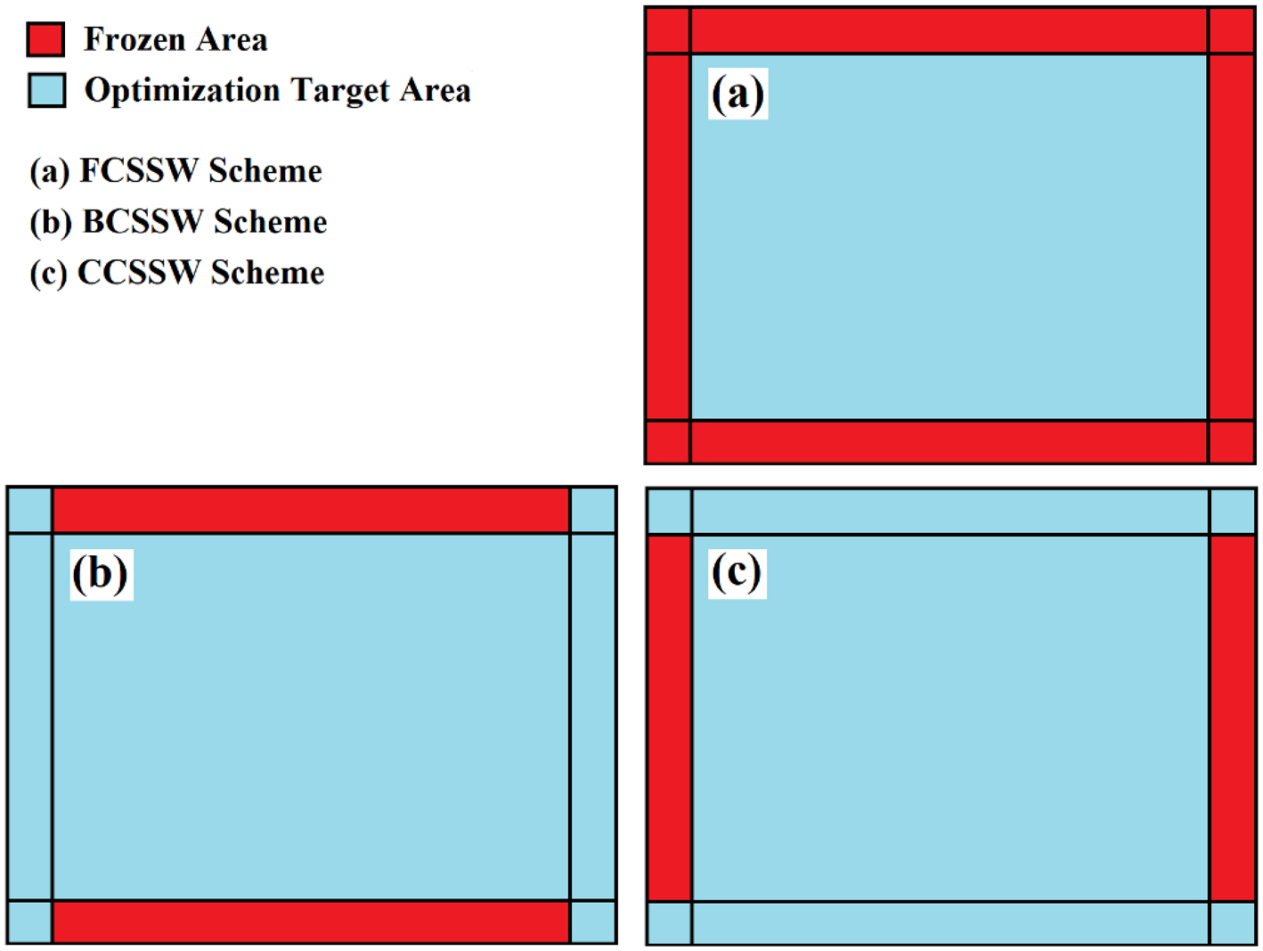
Different zones in the optimization process of the SSWs.

Besides the mentioned consideration above, three other assumptions were also considered in the TO theories. Here, different zones in the infill plate were considered frozen areas, which meant that these areas were out of the optimization scope. The frozen areas and other areas as the targeted zones of the optimization are illustrated in Fig. 3.

After considering the mentioned assumptions, the TO theories together with the finite element analysis software were implemented to optimize the infill plate of the SSW. The procedure of the finite element analysis is briefly described in the next subsection.

To clarify the naming system in the current research paper, the nomenclature of the samples for the finite element analysis and TO results are reported in Table 2.

**Table 2:** Nomenclature of the samples.

Specimen	Full Name
FCSSW-VC60	Fully-Connected Steel Shear Wall-Volume Constraint 60%
FCSSW-VC70	Fully-Connected Steel Shear Wall-Volume Constraint 70%
FCSSW-VC80	Fully-Connected Steel Shear Wall-Volume Constraint 80%
FCSSW-VC90	Fully-Connected Steel Shear Wall-Volume Constraint 90%
BCSSW-VC60	Dominant Beam Connected Steel Shear Wall-Volume Constraint 60%
BCSSW-VC70	Dominant Beam Connected Steel Shear Wall-Volume Constraint 70%
BCSSW-VC80	Dominant Beam Connected Steel Shear Wall-Volume Constraint 80%
BCSSW-VC90	Dominant Beam Connected Steel Shear Wall- olume Constraint 90%
CCSSW-VC60	Dominant Column Connected Steel Shear Wall-Volume Constraint 60%
CCSSW-VC70	Dominant Column Connected Steel Shear Wall-Volume Constraint 70%
CCSSW-VC80	Dominant Column Connected Steel Shear Wall-Volume Constraint 80%
CCSSW-VC90	Dominant Column Connected Steel Shear Wall-Volume Constraint 90%

## Computational field

### Finite Element Analysis (FEA)

Finite Element Analysis (FEA) is the process of modeling objects and systems in a computerized environment with the objective of locating and fixing potential (or existing) structural or performance issues^31^. Preprocessing, which gets the modeling data, processing, which puts the equations together and solves them, and postprocessing, which shows the analysis results, are the three primary steps in the finite element analysis process^32^. The automotive, aerospace, shipbuilding, and construction industries can all benefit greatly from the use of FEA^33^. It provides precise, efficient, and economical answers to engineering problems.

**Figure 4:**
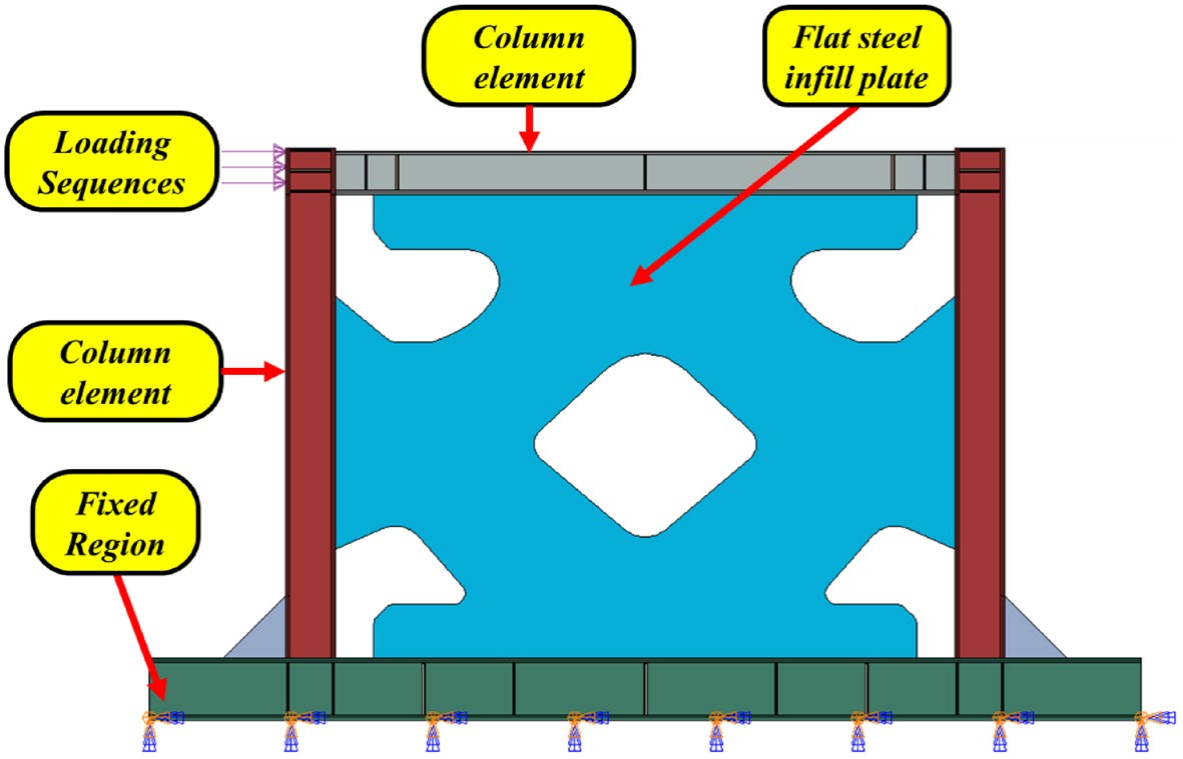
Loading and boundary conditions in the SSWs.

In the current research paper, the SSWs were modeled in the FEA software (Abaqus CAE) and then, the mechanical properties according to Table 3 were assumed as the material properties of the elements. After assembling the elements together, three different solvers were used; nonlinear static and dynamic explicit. It is noteworthy that in the current research paper, to obtain the optimized shape of the infill plate, the TOSCA^34^ plugin was used. Where the mentioned solvers were implemented to obtain the pushover curves, and hysteresis behavior, respectively. The loading and boundary conditions were applied based on Fig. 4 in the optimized SSWs.

**Table 3:** Material properties for three elements in the SSW for the FEA.

Element	Young’s modulus (GPa)	Yield stress, σ_Y_ (MPa)	Ultimate stress, σ_U_ (MPa)	σ_Y_/σ_U_	Elongation (%)
Plate	210	207	290	0.71	41
Column	210	300	443	0.67	33
Beam	210	288	456	0.63	37

**Figure 5:**
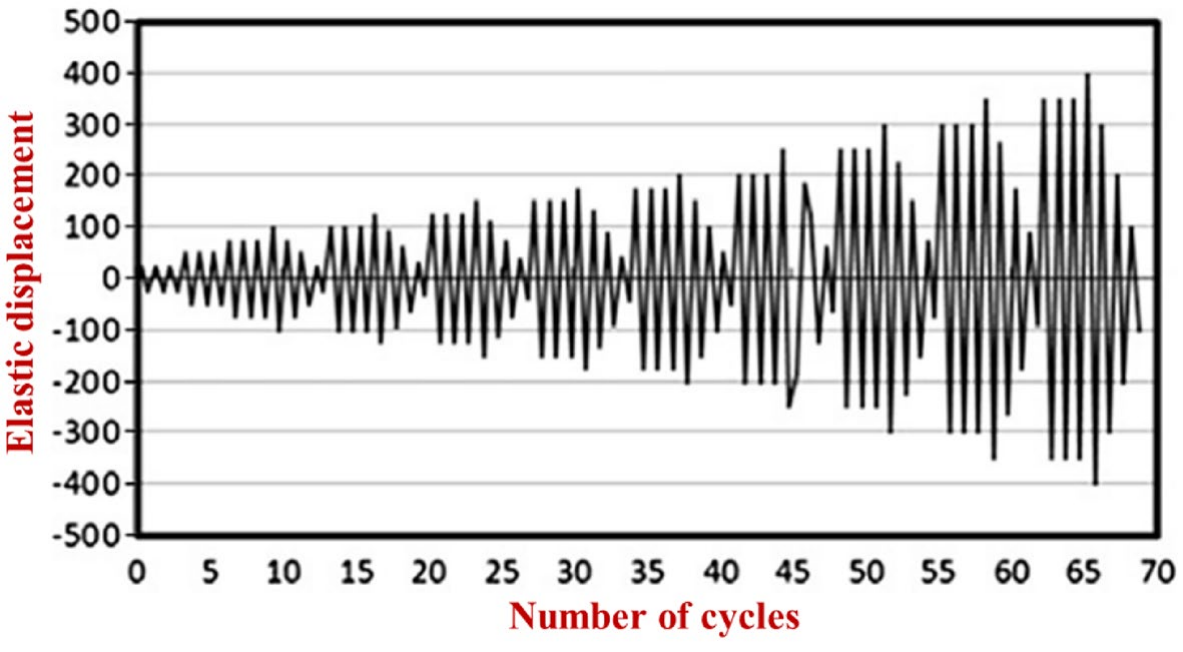
Loading sequences that were applied to the optimized SSWs in the FEA^28^.

The loading sequences were applied to the optimized SSWs according to Fig. 5. This loading condition presents seismic loading sequences like earthquakes.

**Figure 6:**
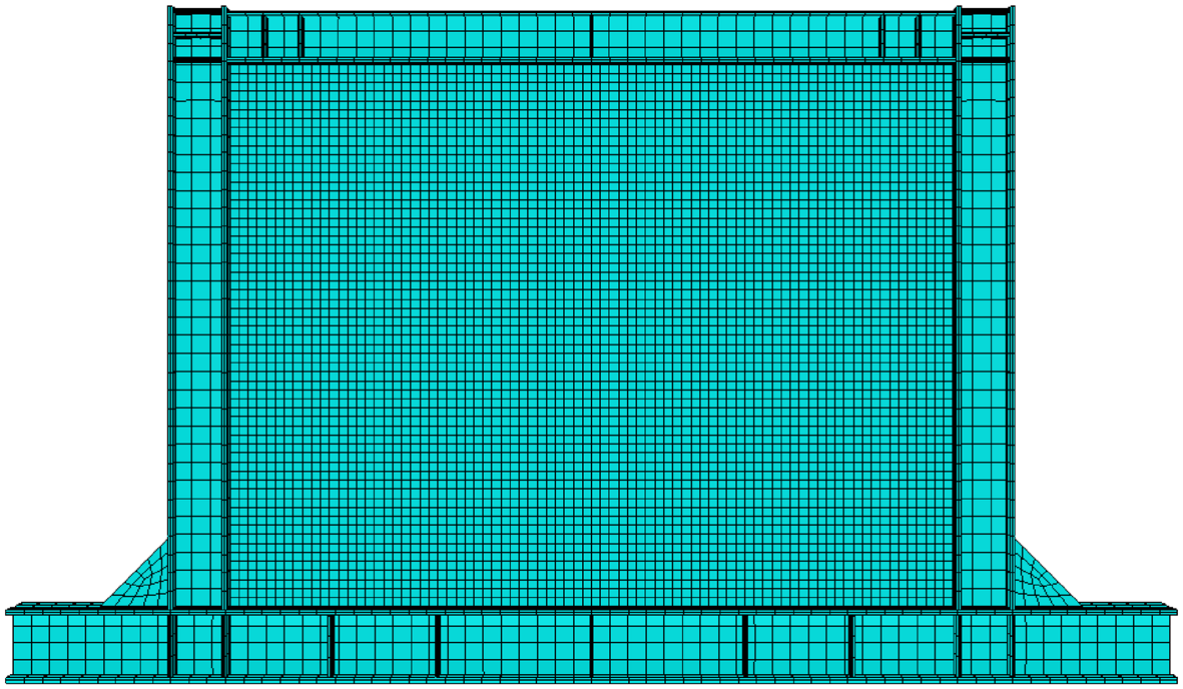
Mesh pattern in the SSW with the flat infill plate.

**Figure 7:**
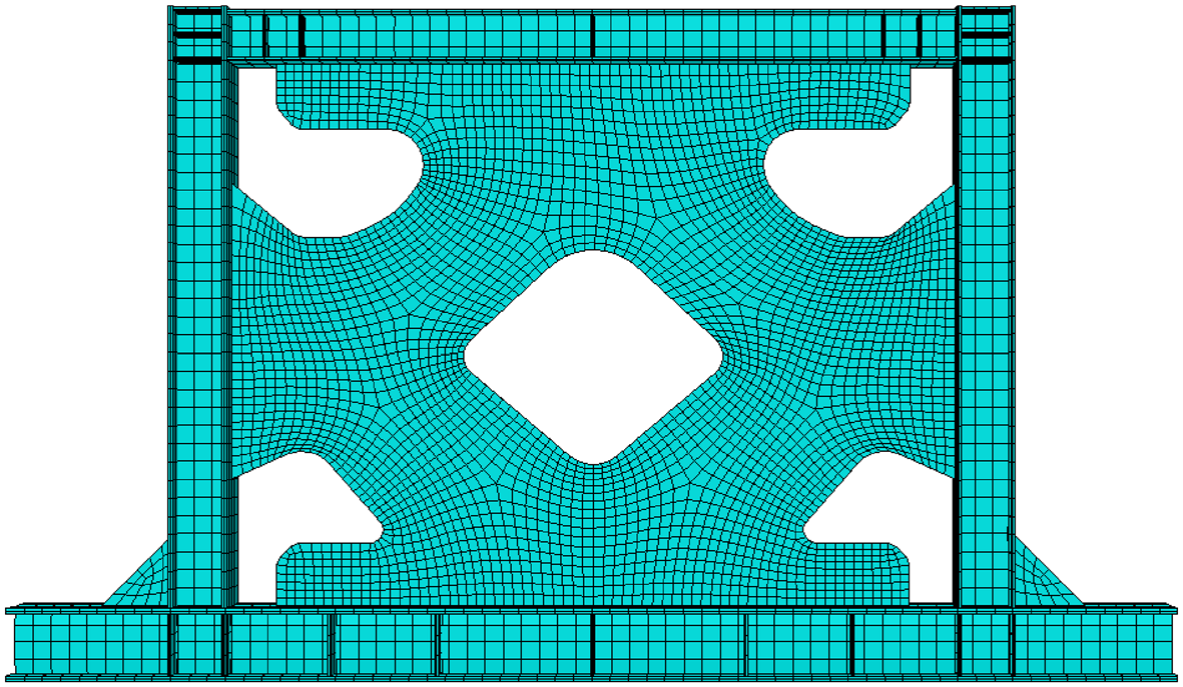
Mesh pattern in the SSW with an optimized infill plate.

The designed shear walls were meshed apart following the aforementioned processes (see Fig. 6). Also, after performing the TO procedures and implementing the mentioned plugin in the FEA, the optimized SSWs were also meshed apart. Figure 7 shows a representative illustration of the SSW with an optimized infill plate. The mesh convergence analysis was also used to determine the appropriate number and sizes of elements for the shear walls, with the element type being S8R that is based on the reduced integration method and with the order of two. In the end, a 5 cm mesh size was used for the frame parts and a 2.5 cm mesh size for the infill plate.

### Validation study

**Figure 8:**
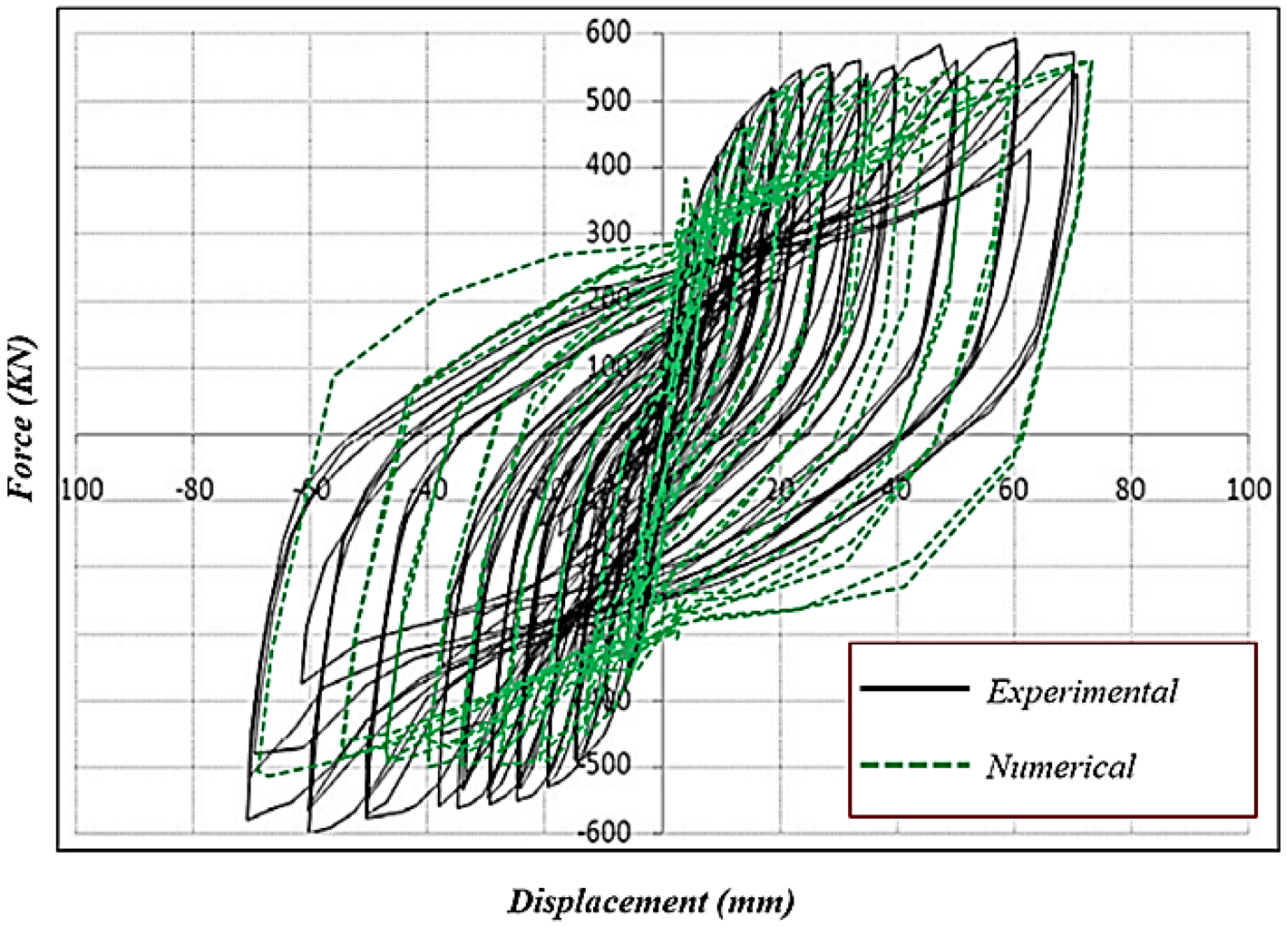
Hysteresis curves of the flat SSW that were obtained numerically and experimentally^28^.

**Figure 9:**
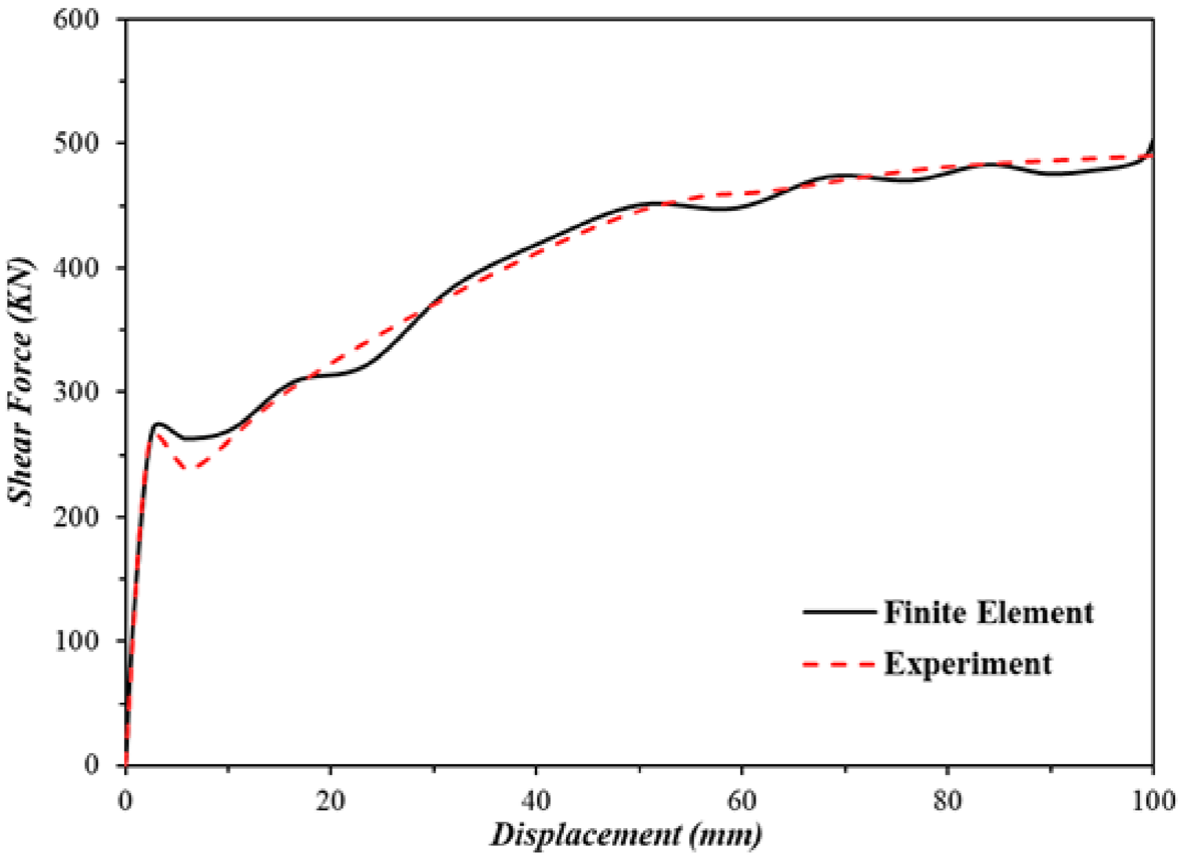
Comparison of the pushover curves that were evaluated experimentally^28^ and numerically.

Any FEA model initially should be verified by some experimental results. This issue confirms the applicability of the modeled samples. The modeled samples in the FEA section of this paper were validated by the results reported by Emami et al.^28^. For this aim, the hysteresis and pushover curves that were obtained numerically here were compared to those reported by the experimental study^28^. The hysteresis behavior and pushover performance of the flat SSW are presented in Figs. 8 and 9, respectively. Based on these figures, the modeled samples in the current research paper could predict the SSWs’ performance with minor errors.

## Results and discussions

### Recommended infill plates (optimized)

**Figure 10:**
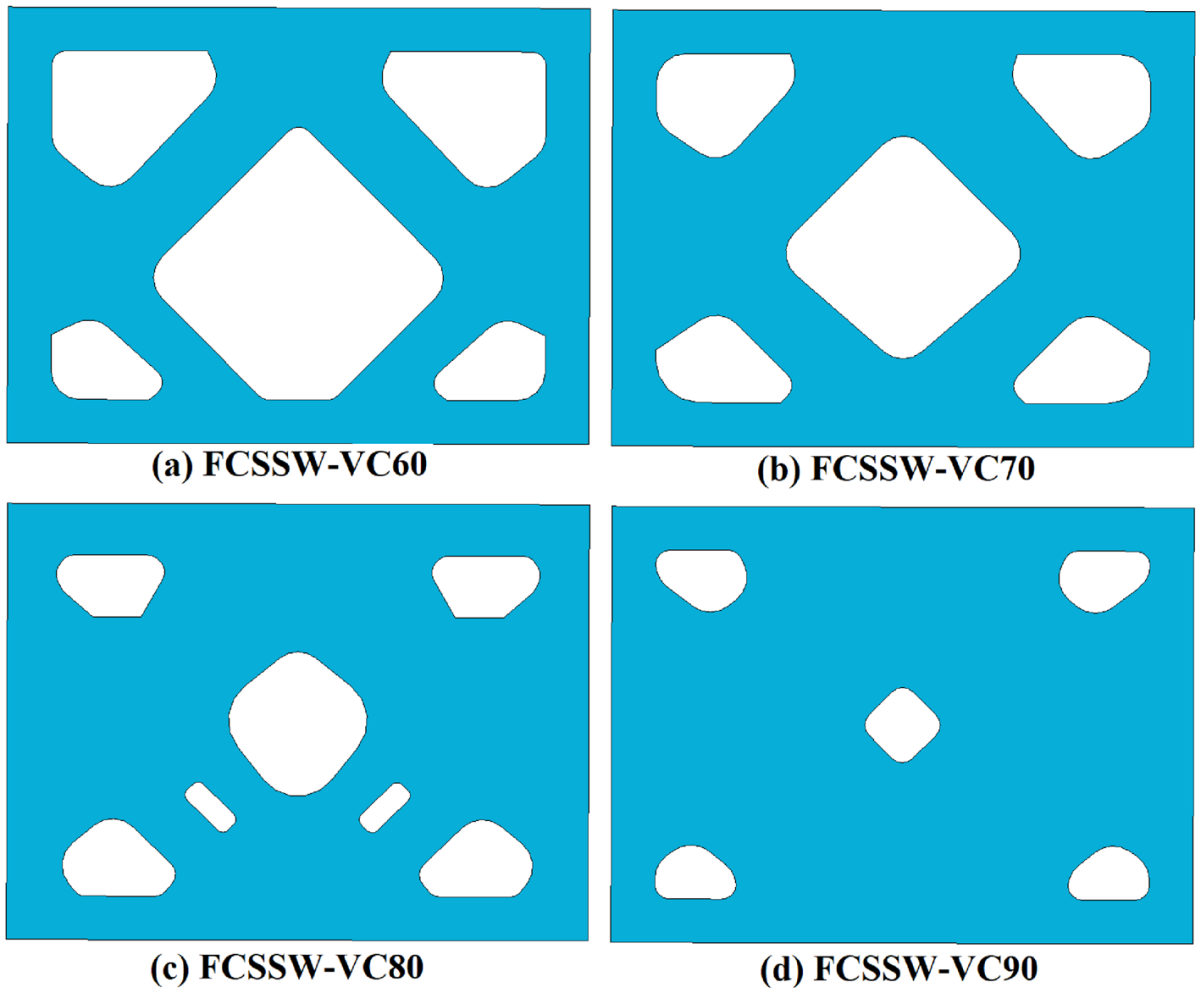
The optimized infill plates of FCSSW samples.

**Figure 11:**
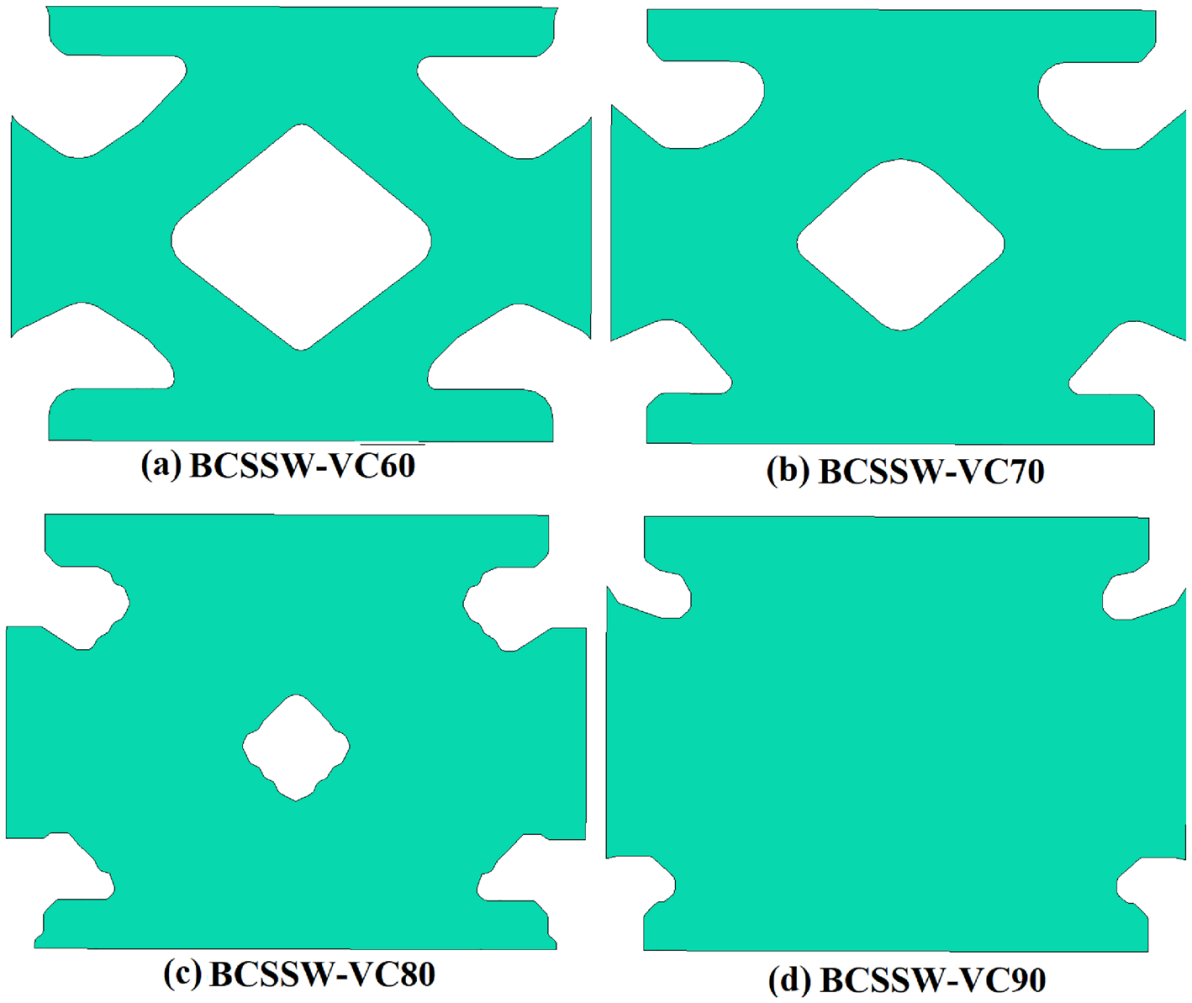
The optimized infill plates of BCSSW sample.

**Figure 12:**
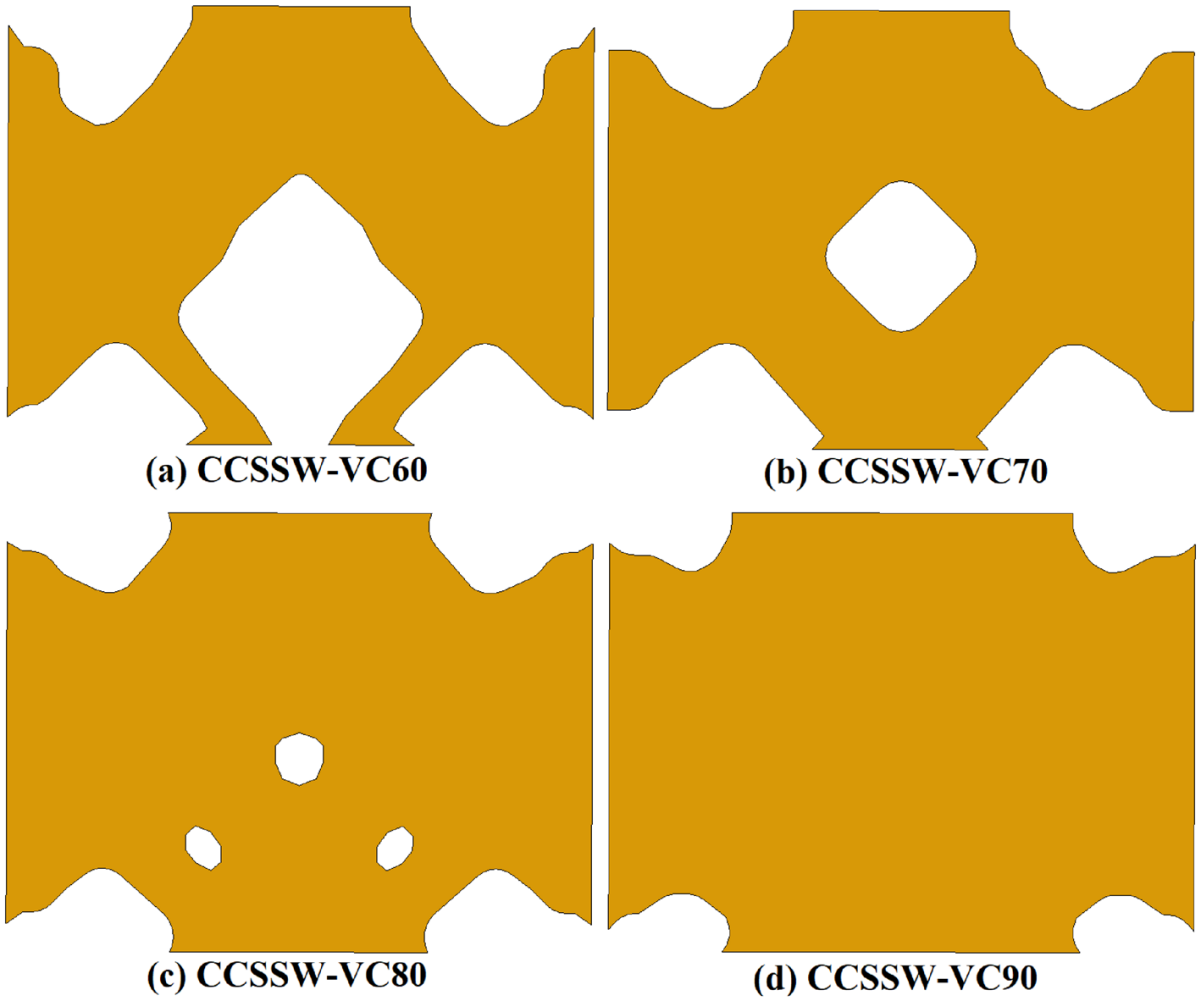
Infill plates of optimized CCSSW sample.

According to Fig. 2, three different conditions were assumed for the topology optimization besides, four volume constraints of 60%, 70%, 80%, and 90% together with the minimization of the strain energy were considered. Based on the stated approaches in section “Methodology” and FEA procedures the recommended and optimized infill plates are discussed in the current section. The optimized SSWs with the mentioned assumptions are presented in Figs. 10, 11, and 12 for the samples of FCSSW, BCSSW, and CCSSW, respectively. The volume constraints for each sample are also represented in the mentioned figures.

After obtaining the optimized SSWs and showing them in the above figure, the cyclic performance of them was evaluated to see the mentioned SSWs’ operating quality against the seismic loading sequences. The next subsection contains the obtained results and discussions about the reasons.

### Stress distribution

**Figure 13:**
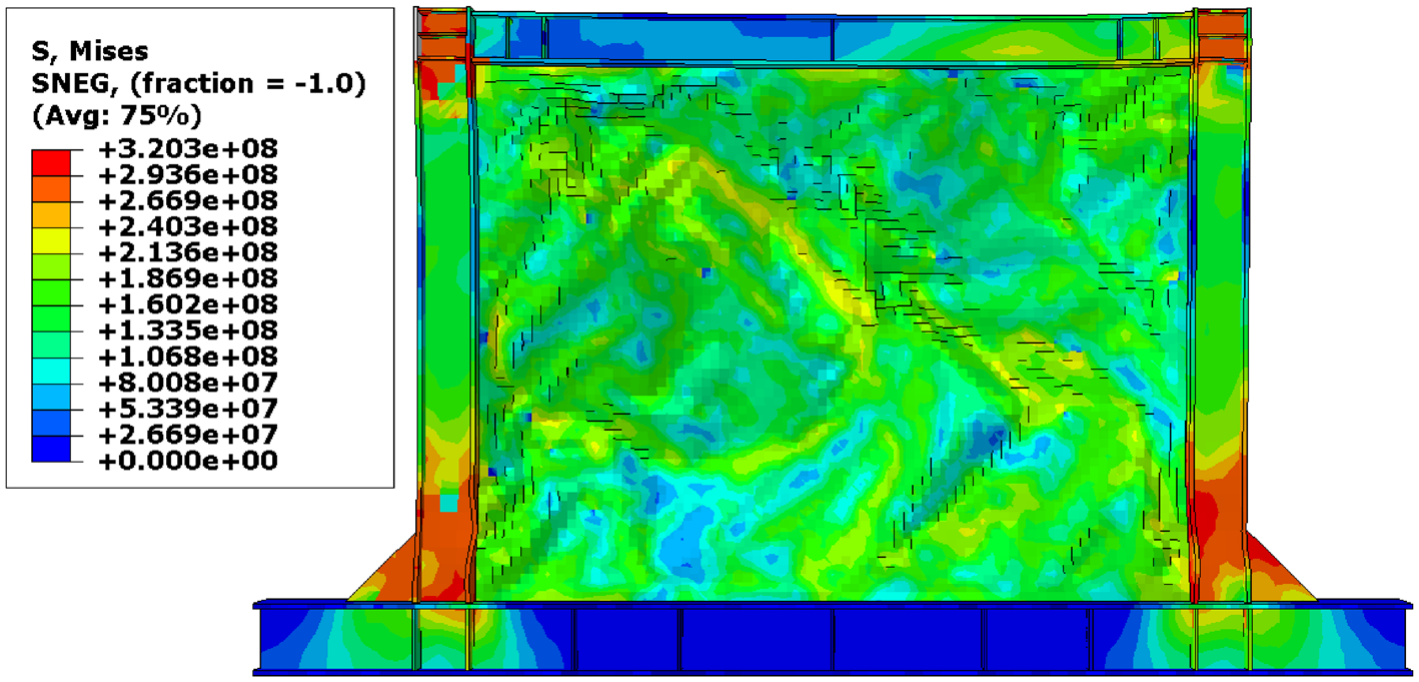
Stress distribution in the flat SSW.

**Figure 14:**
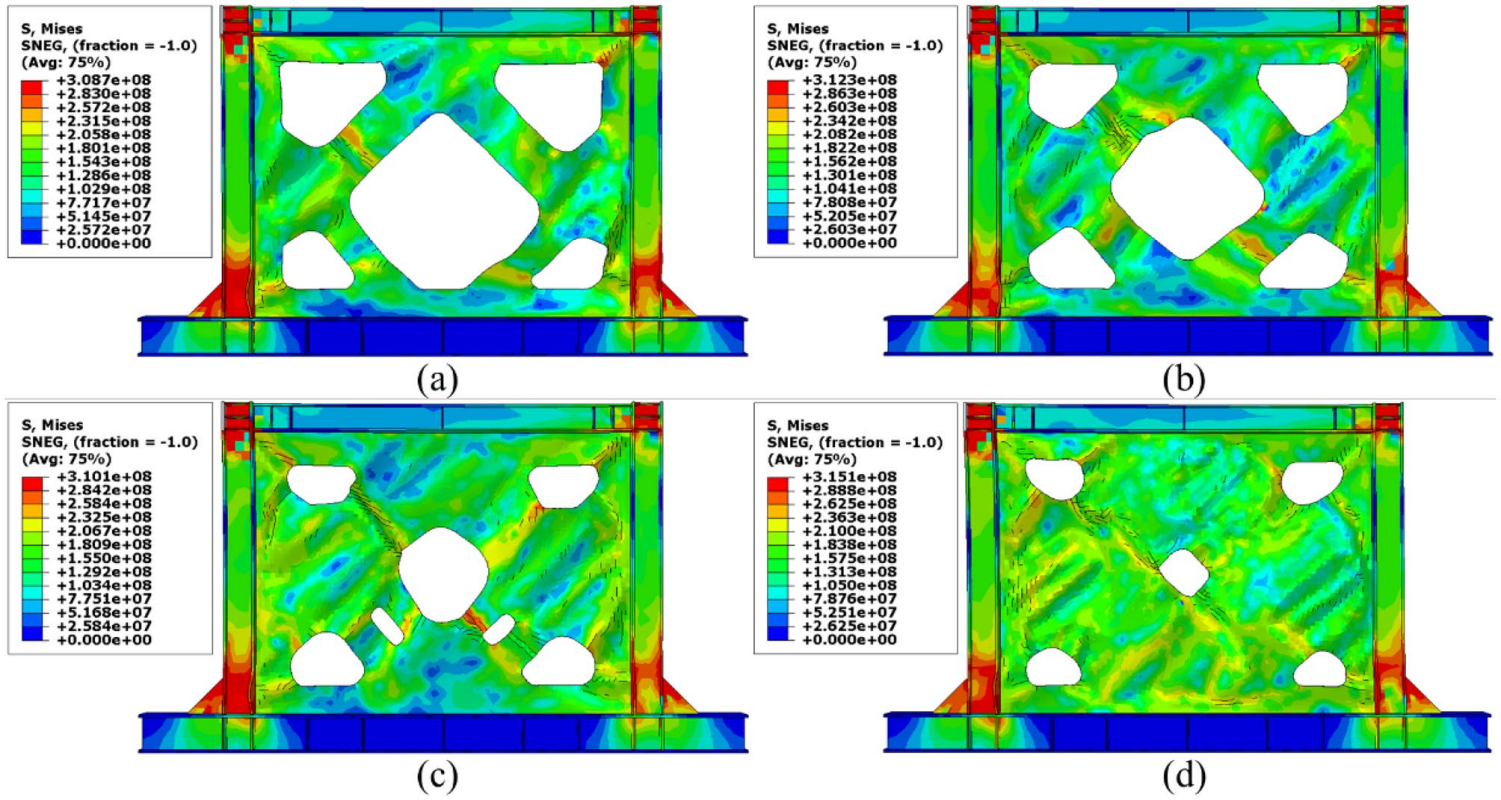
Stress distribution in the FCSSW configuration with volume constraint of (**a**) 60%, (**b**) 70%, (**c**) 80%, and (**d**) 90%.

**Figure 15:**
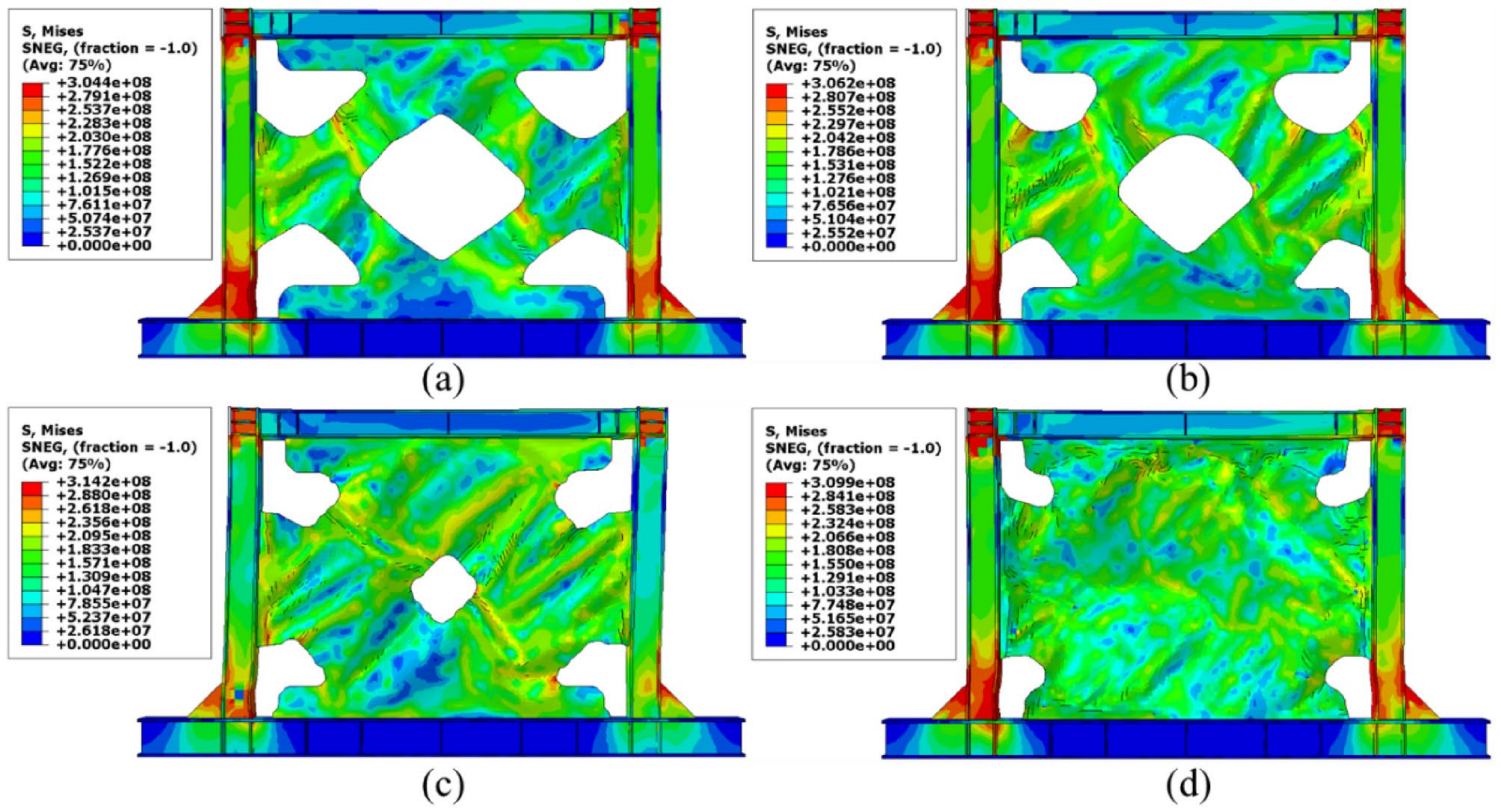
Stress distribution in the BCSSW configuration with volume constraint of (**a**) 60%, (**b**) 70%, (**c**) 80%, and (**d**) 90%.

**Figure 16:**
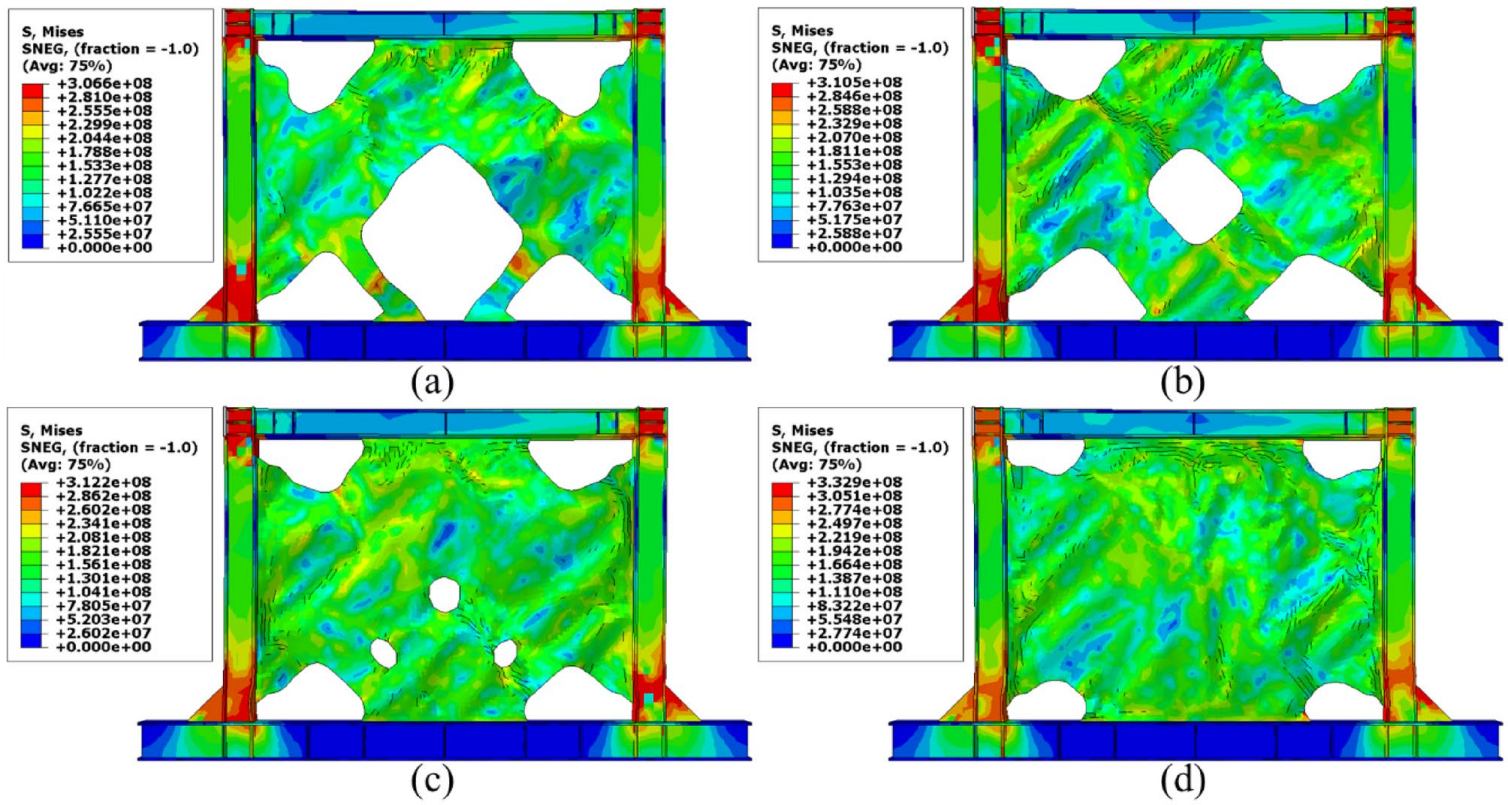
Stress distribution in the CCSSW configuration with volume constraint of (**a**) 60%, (**b**) 70%, (**c**) 80%, and (**d**) 90%.

The von-Mises stress distributions in the different configurations of the SSWs are illustrated in this subsection. Figure 13 shows the stress distribution in the SSW with the flat infill plate. Based on this figure, the corners where the infill plate joined to the other elements resulted in higher amounts of stress. Thus, these zones are known as the stress concentration zones. Figures 14, 15, and 16 present the stress distributions in the FCSSW, BCSSW, and CCSSW configurations, respectively. The TO procedure could reduce the amounts of stress in the stress concentration zones and also, helped to good distribution of the stress over the infill plate of the SSWs. This claim was completely approved at the bottom of the columns of the optimized SSWs in Figs. 14 and 15.

### Plastic strain distribution

**Figure 17:**
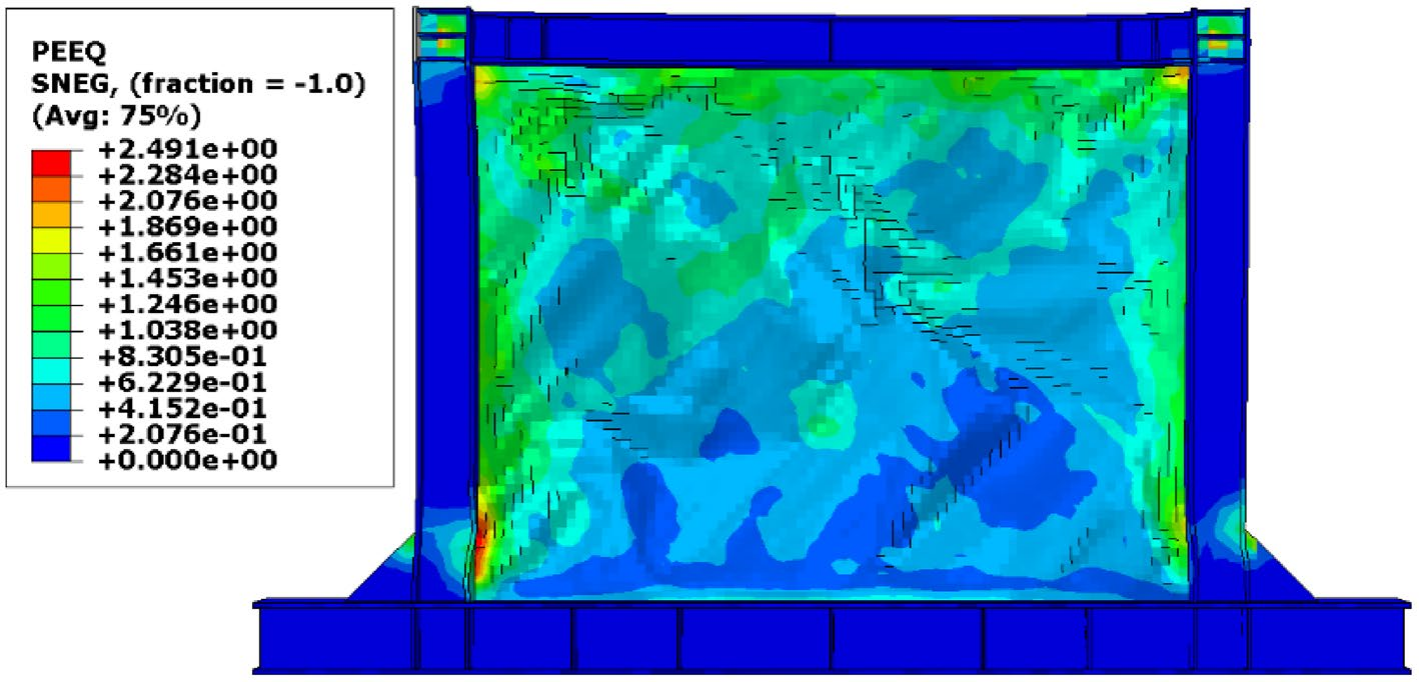
Plastic strain distribution in the SSW with the flat infill plate.

**Figure 18:**
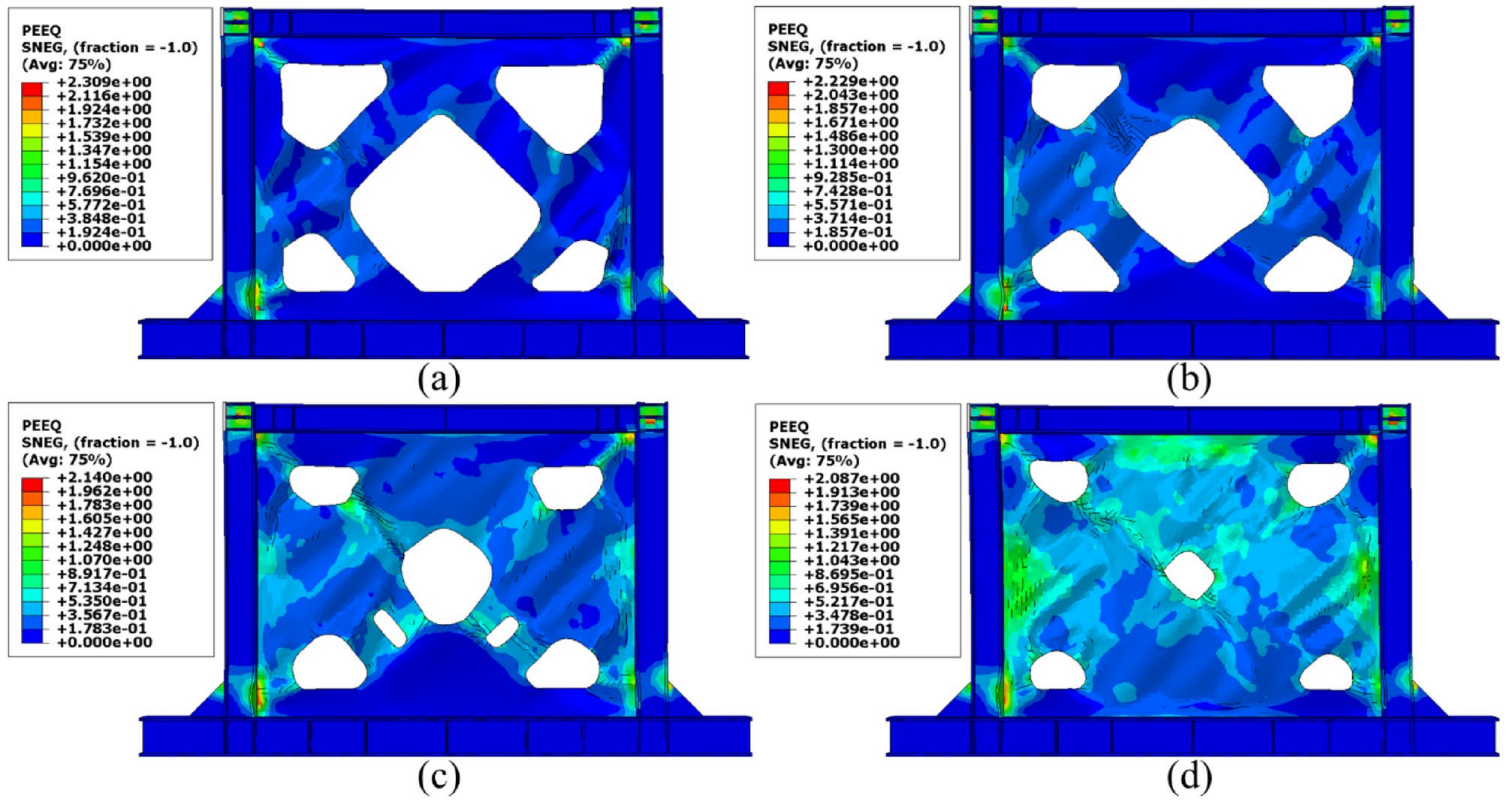
Plastic strain distribution in the FCSSW configuration with the volume constraint of (**a**) 60%, (**b**) 70%, (**c**) 80%, and (**d**) 90%.

**Figure 19:**
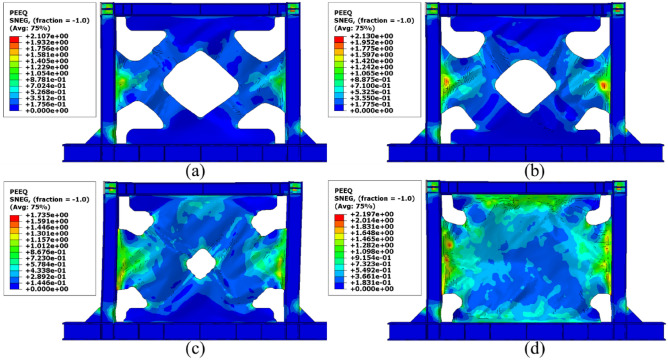
Plastic strain distribution in the BCSSW configuration with the volume constraint of (**a**) 60%, (**b**) 70%, (**c**) 80%, and (**d**) 90%.

**Figure 20:**
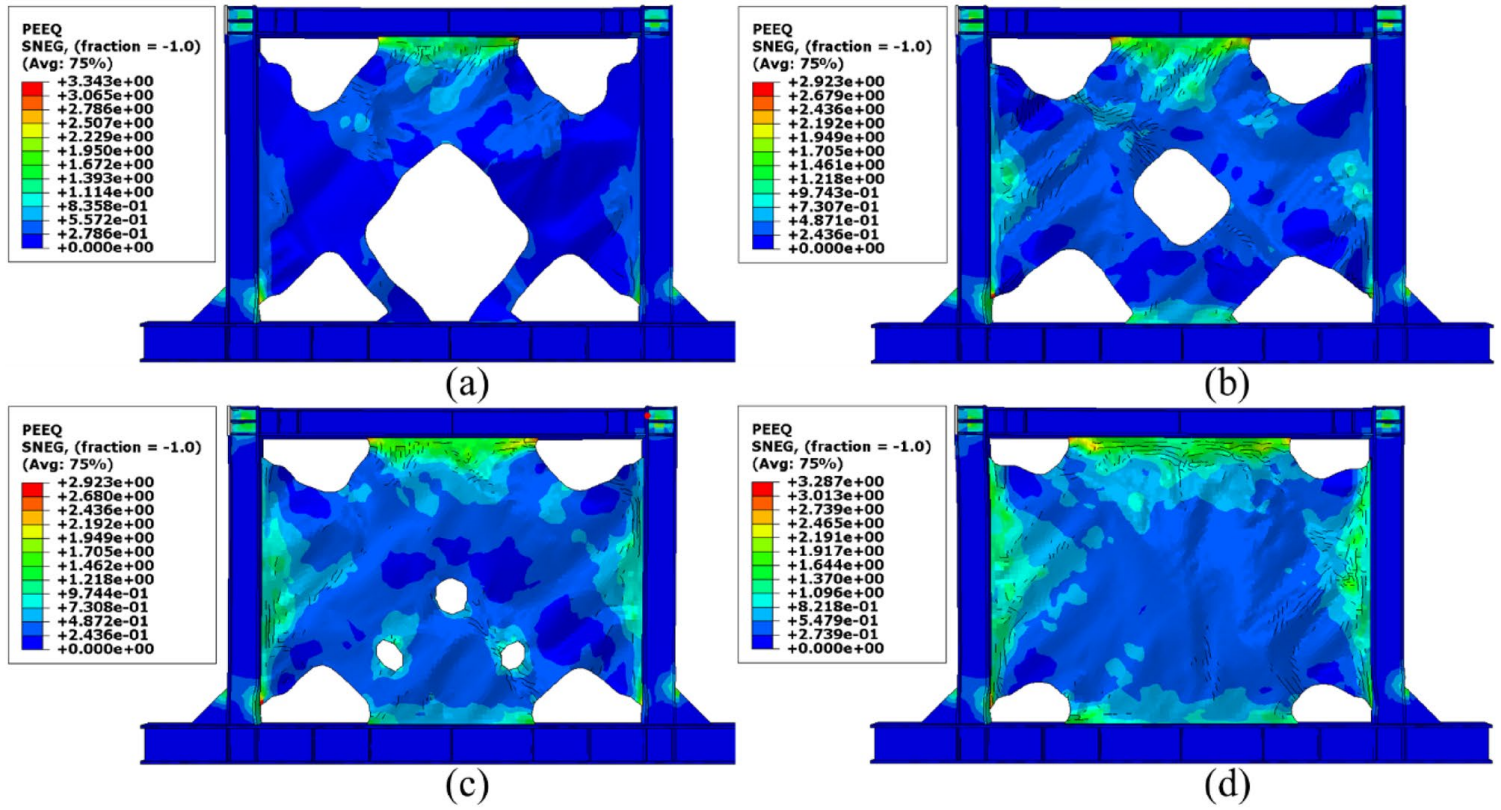
Plastic strain distribution in the CCSSW configuration with the volume constraint of (**a**) 60%, (**b**) 70%, (**c**) 80%, and (**d**) 90%.

Besides the stress distribution, the plastic strain distribution obtained from the FEA can be helpful because in most cases, higher plastic strains can result in structure collapsing. To this end, the plastic distributions in different SSWs are illustrated in the current section. Figure 17 shows the plastic strain values and their distributions in the SSW with a fully flat infill plate. Based on this figure, the corners of the SSW experienced higher plastic strains compared to the other vicinities in the SSW. However, using topology optimization resulted in distributing the plastic strains all over the infill plate. Figures 18, 19, and 20 relate to the SSWs with configurations of FCSSW, BCSSW, and CCSSW, respectively. Two configurations of FCSSW and BCSSW could reduce the amounts of plastic distributions in the SSW and the lowest amount related to the 80% volume constraint. The CCSSW configuration (based on Fig. 20) increased the plastic strain distributions’ amounts, which meant that these SSWs experienced more plastic strains and consequently more plastic deformation. Thus, the CCSSW could absorb more exerted energies by utilizing plastic deformations. One of the consequences of the mentioned issue was the out-of-plane displacements which are discussed extensively in the next subsection.

### Out-of-plane displacement

**Figure 21:**
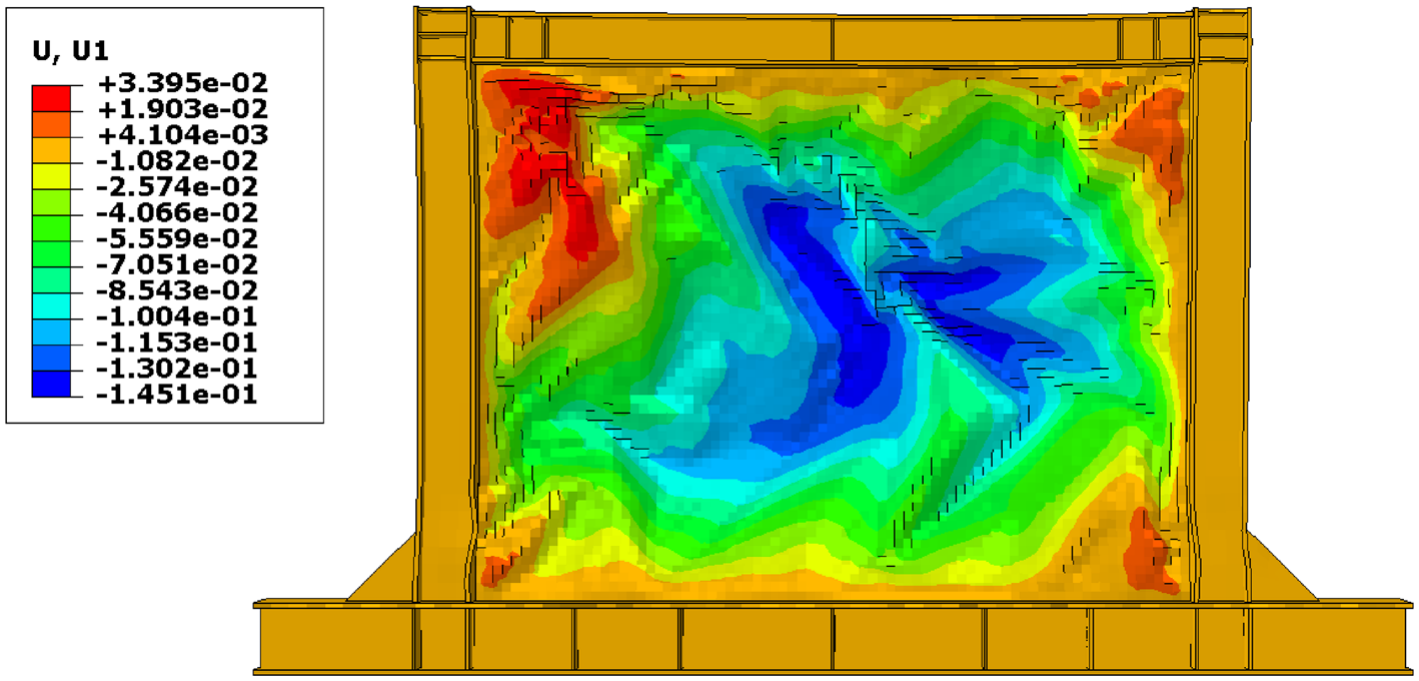
Out-of-plane displacement distribution in the SSW with the flat infill plate.

**Figure 22:**
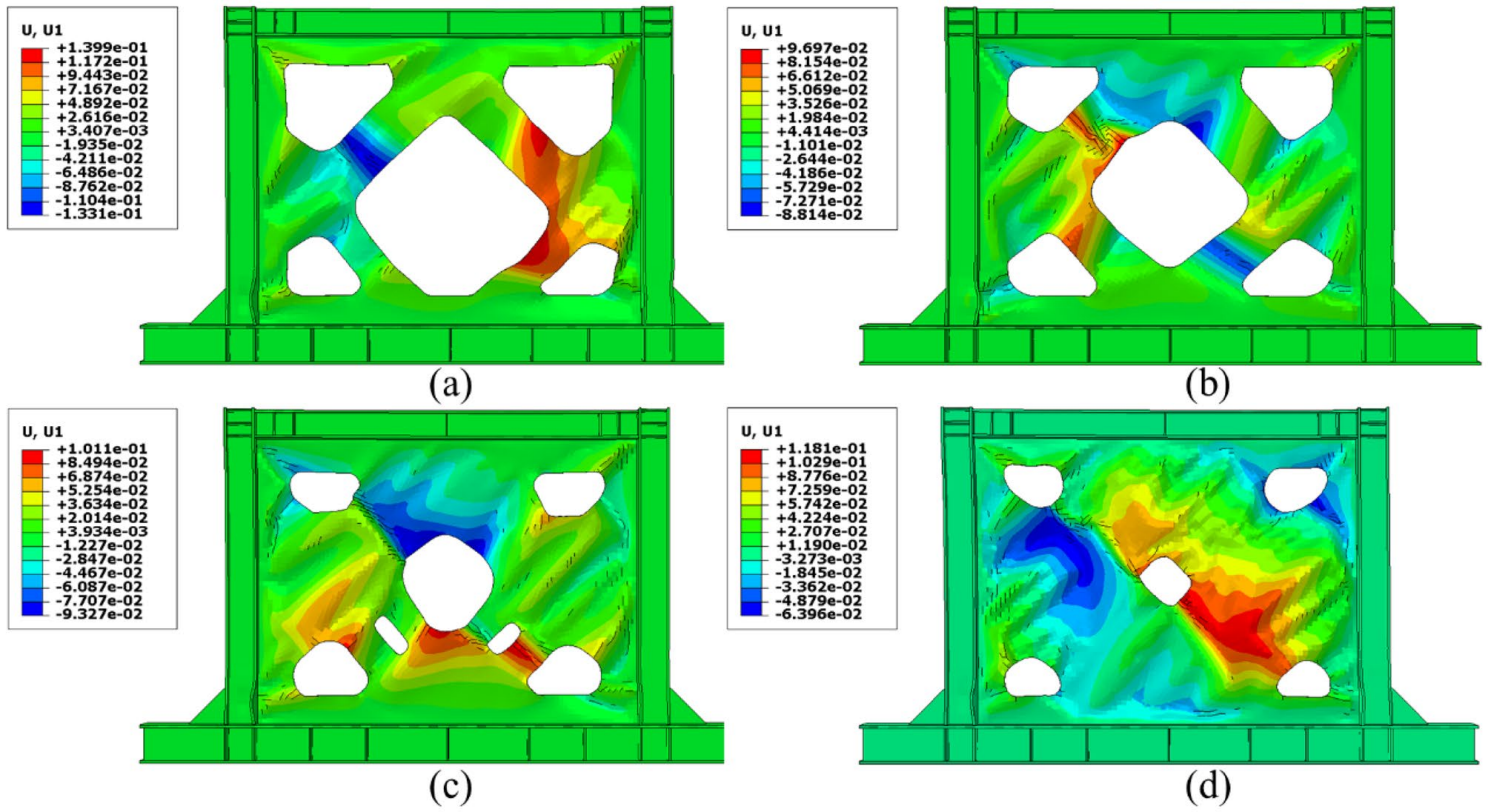
Out-of-plane displacement distribution in the FCSSW configuration with the volume constraint of (**a**) 60%, (**b**) 70%, (**c**) 80%, and (**d**) 90%.

**Figure 23:**
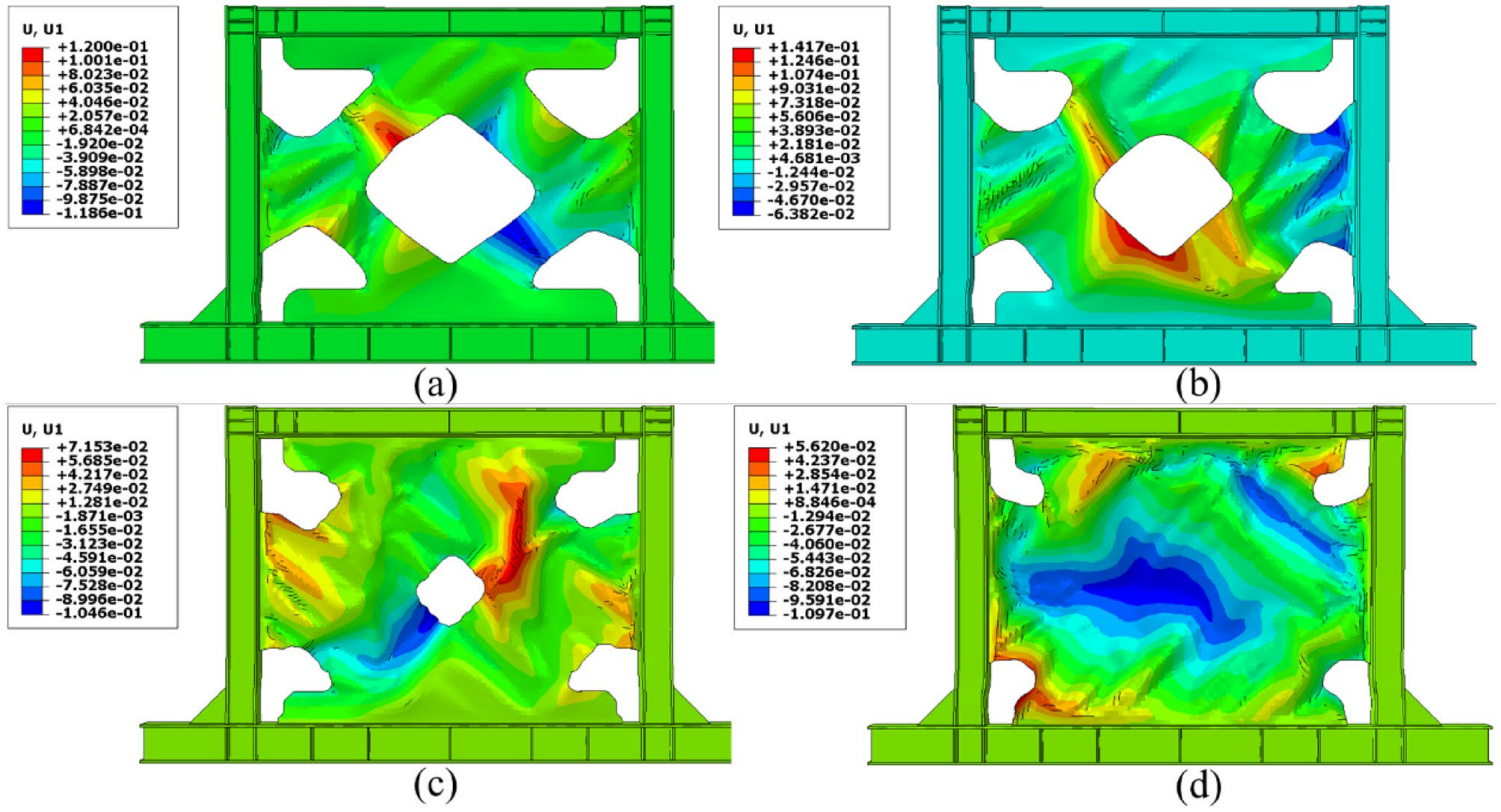
Out-of-plane displacement distribution in the BCSSW configuration with the volume constraint of (**a**) 60%, (**b**) 70%, (**c**) 80%, and (**d**) 90%.

**Figure 24:**
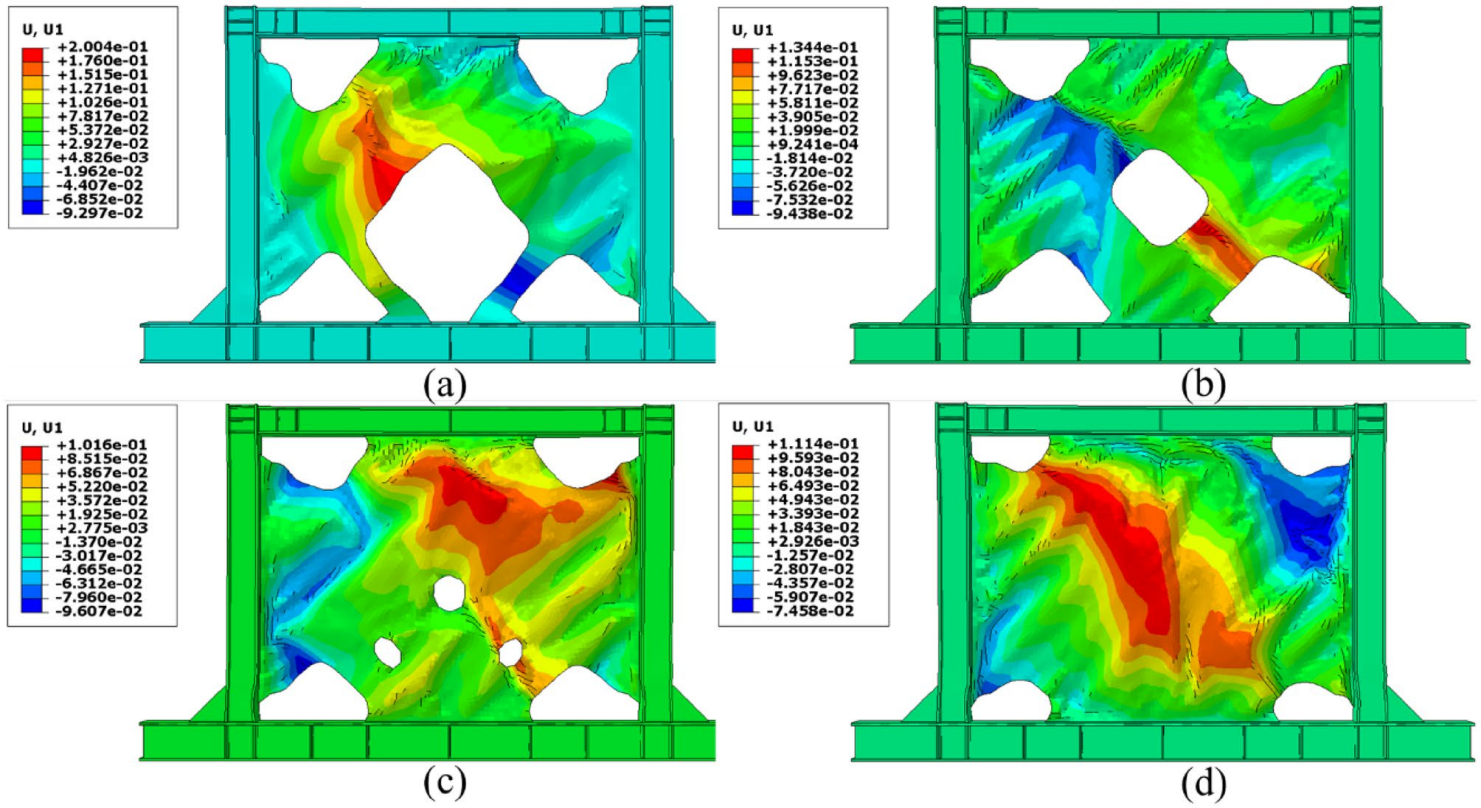
Out-of-plane displacement distribution in the CCSSW configuration with the volume constraint of (**a**) 60%, (**b**) 70%, (**c**) 80%, and (**d**) 90%.

One of the most important challenges that the shear walls face is the high out-of-plane displacement. Thus, controlling this issue can be helpful to the seismic performance of the shear walls. The out-of-plane distributions that were evaluated from the FEA are presented in Figs. 21, 22, 23, and 24 for the SSWs’ configurations of flat infill plate, FCSSW, BCSSW, and CCSSW, respectively. Comparing the obtained results in these figures revealed that the highest values of out-of-plane displacement related to the fully flat infill plate SSW (see Fig. 21). Implementing the TO could reduce these values, thus, the optimized SSW experienced lower out-of-plane displacement. Hence, the optimized SSW could be more applicable and safer in comparison to the simple SSW from a seismic loading condition point of view. Considering the volume constrained that were assumed in the TO procedure, the 80% and 90% volume constraints could present lower out-of-plane displacement, thus, these two constraints could be more reliable than the other two volume constraints.

It should be noted that in some cases, the 60% and 70% volume constraints resulted in higher out-of-plane displacements compared to the flat SSW that is exhibited in Fig. 21.

### Hysteresis curves

**Figure 25:**
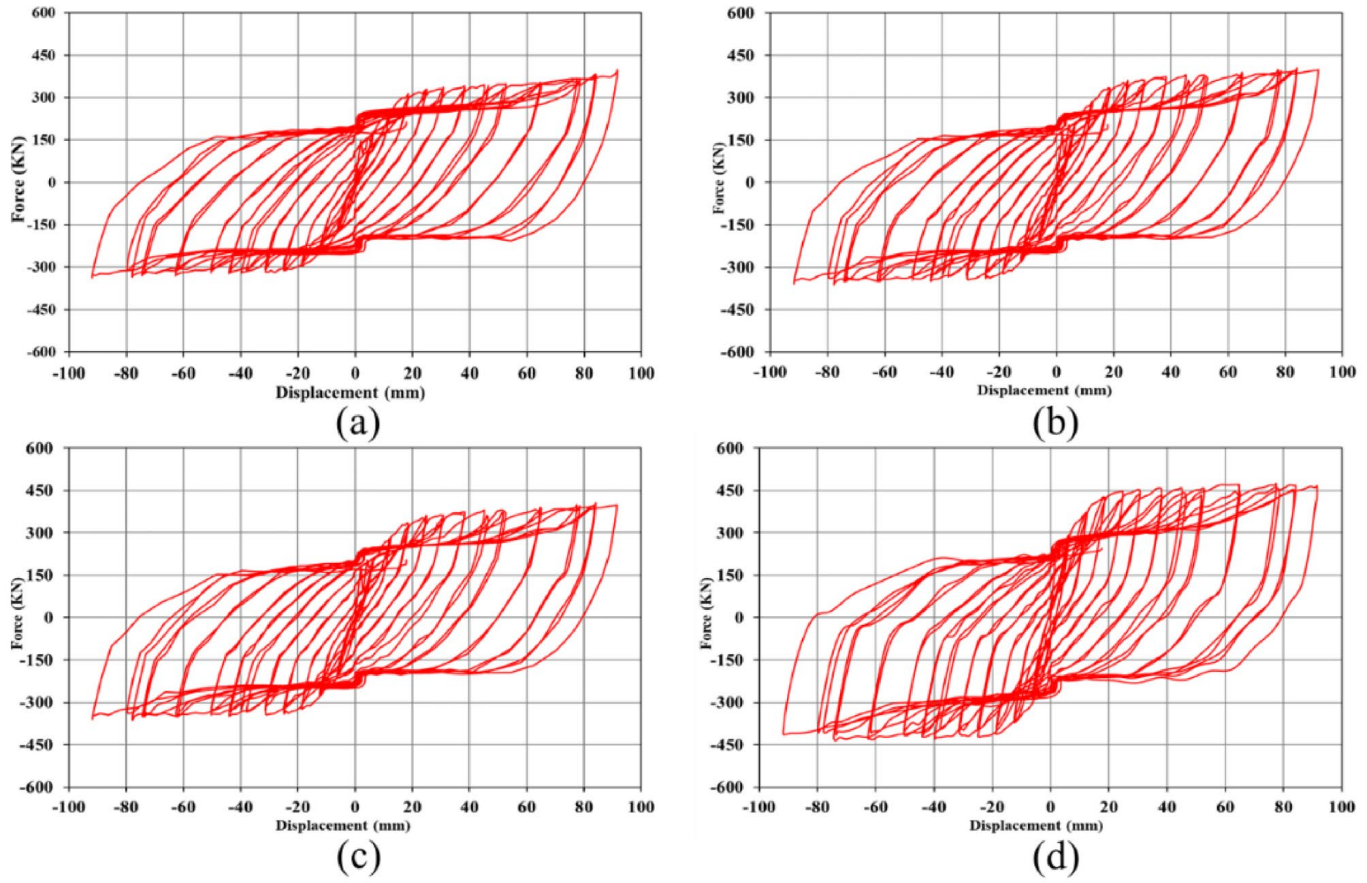
Hysteresis curves of FCSSW configuration with the volume constraint of (**a**) 60%, (**b**) 70%, (**c**) 80%, and (**d**) 90%.

**Figure 26:**
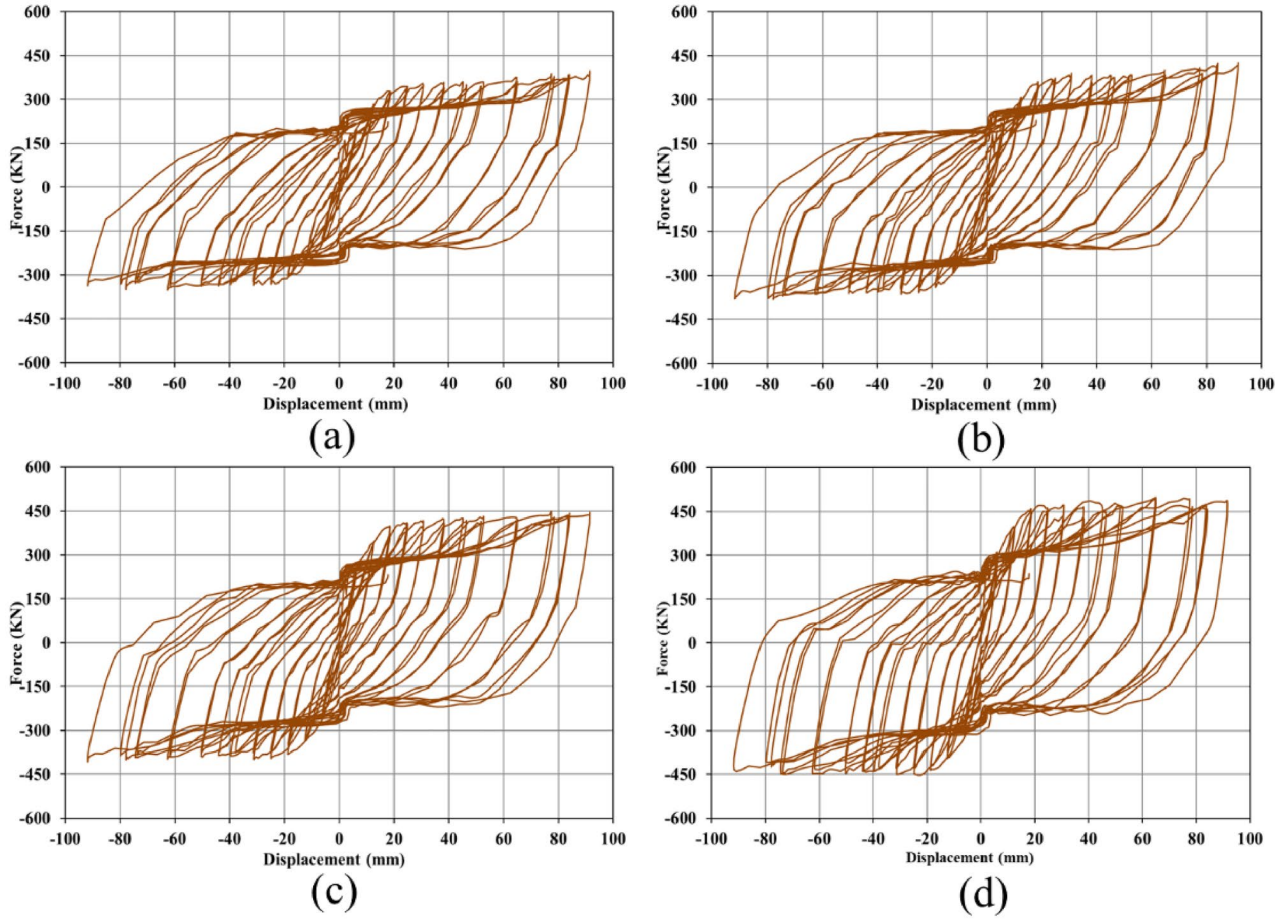
Hysteresis curves of BCSSW configuration with the volume constraint of (**a**) 60%, (**b**) 70%, (**c**) 80%, and (**d**) 90%.

**Figure 27:**
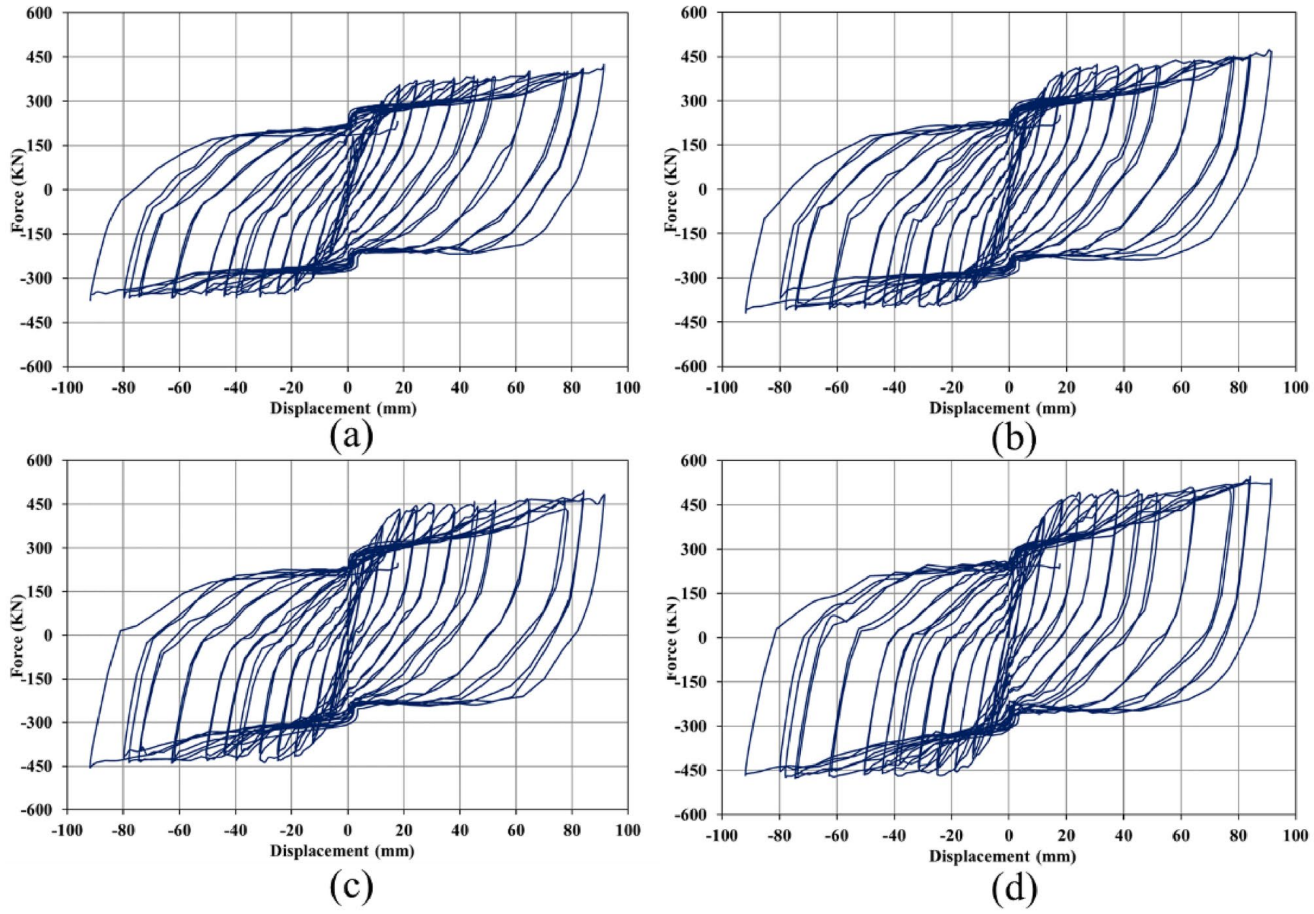
Hysteresis curves of CCSSW configuration with the volume constraint of (**a**) 60%, (**b**) 70%, (**c**) 80%, and (**d**) 90%.

When a loading sequence is applied to a structure cyclically, the mentioned structure experiences the hysteresis behavior. In the current research paper, the seismic loading condition was applied according to the curve plotted in Fig. 5, and then, the hysteresis responses of the SSWs were recorded in the FEA and plotted in a single curve. As stated earlier in the FEA section, the loading condition that was applied to the SSWs was displacement control, thus, the hysteresis curves presented constant values of displacement but the evaluated forces were different. The hysteresis curves of SSW with various configurations of FCSSW, BCSSW, and CCSSW are presented in Figs. 25, 26, and 27, respectively.

The hysteresis curves are loops and they have an inside area which means the absorbed energy by the SSWs. The hysteresis curve of a flat SSW is represented in Fig. 8. Considering this figure with those presented in the current subsection, confirmed that topology optimization could not expand the loops of hysteresis curves and consequently the absorbed exerted energies by the cyclic loading sequences. The highest value of the exerted force in the flat SSW (shown in Fig. 8) was around 600 KN but the optimized SSWs with three different configurations of FCSSW, BCSSW, and CCSSW and volume constraints resulted in lower forces.

Comparing the presented results gave a better inside into the seismic performance of the various types of studied SSWs. In each category of the SSW, the 80% and 90% volume constraints especially the 90% could result in the higher area inside the hysteresis loop in comparison to the other ones. As a matter of fact, the 90% volume constraint was similar to the 100% one (the fully flat SSW that was exhibited in Fig. 8) but the only problem with this volume constraint was the higher weight and costs of fabrication processes. However, in a single case of volume constraint (e.g., 90%) the CCSSW configuration of SSW presented the best response from a hysteresis curve point of view. The main reason for this issue was the frozen area in the TO procedure because, in a shear wall, the columns play a critical role in controlling the structure strength.

The other parameter, strength, was computed and plotted in a curve with the variation in drift angle (i.e., Strength-Drift Angle curves) after the hysteresis curves of the various shear walls were recorded. Each hysteresis curve’s peak loads were chosen, combined, and given a strength designation. Additionally, the drift angle was named after dividing the horizontal displacement by the shear wall column’s length. Plotted strength-drift angle curves for various shear wall configurations, including the Simple shear wall, are displayed here.

**Figure 28:**
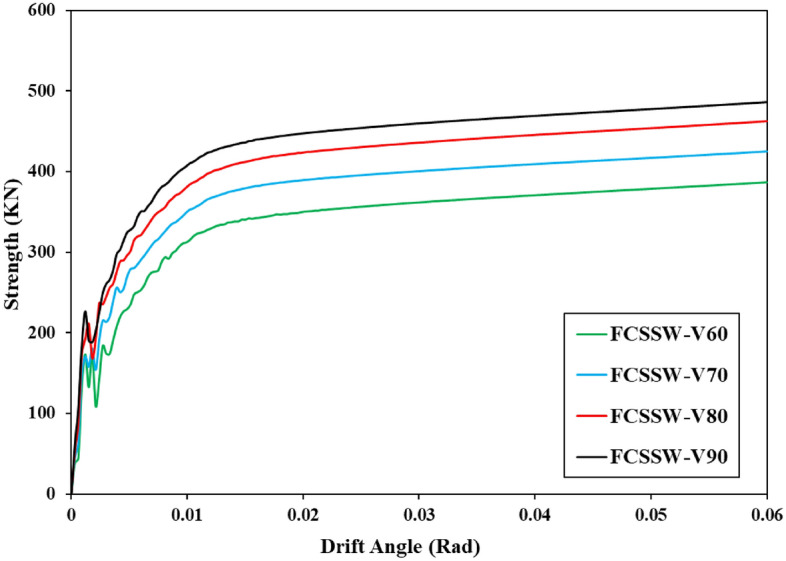
Strength-drift angle for the FCSSW configuration with various volume constraints.

**Figure 29:**
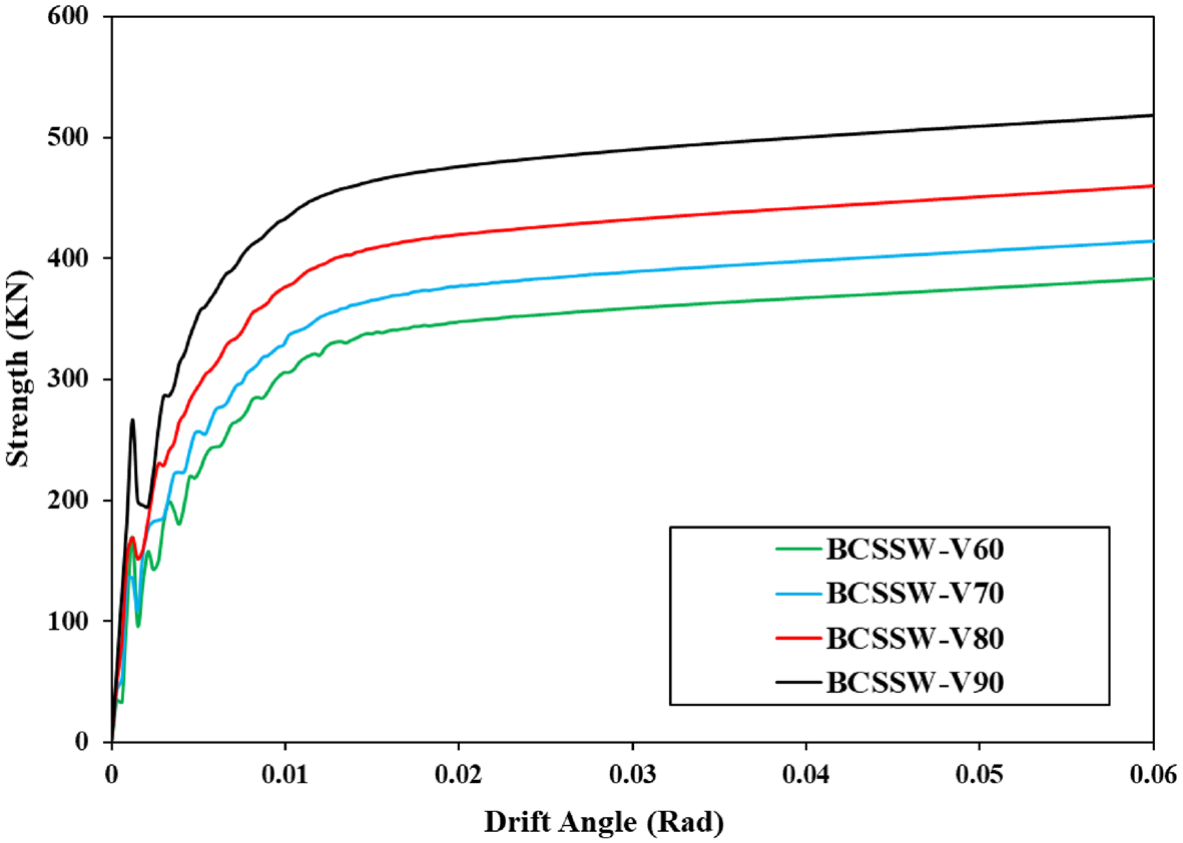
Strength-drift angle for the BCSSW configuration considering various volume constraints.

**Figure 30:**
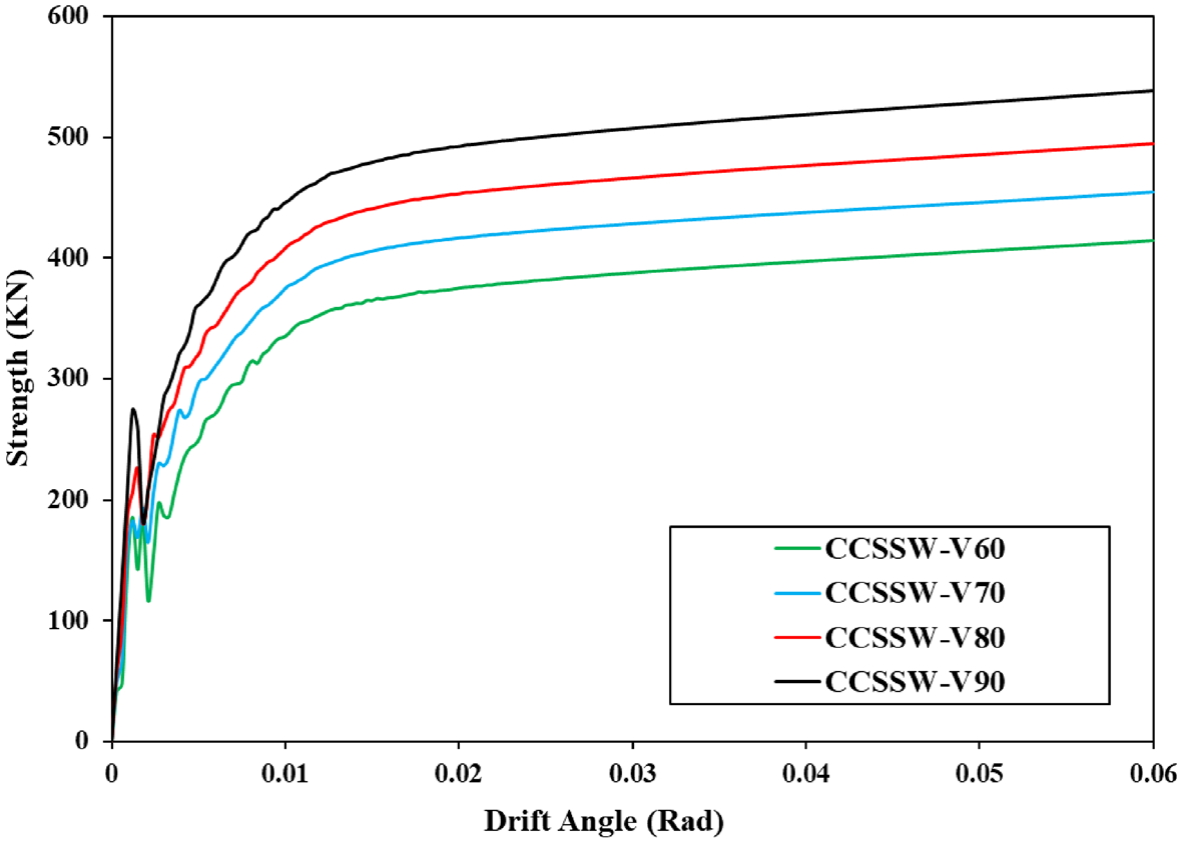
Strength-drift angle of the CCSSW configuration with different infill patterns.

The mentioned results are plotted in the shown curves of Figs. 28, 29, and 30. These curves also confirmed that the volume constraint of 90% had the highest value of strength and also, the CCSSW configuration of SSW was the best optimized SSW compared to the other two systems.

### Cumulated energy

**Figure 31:**
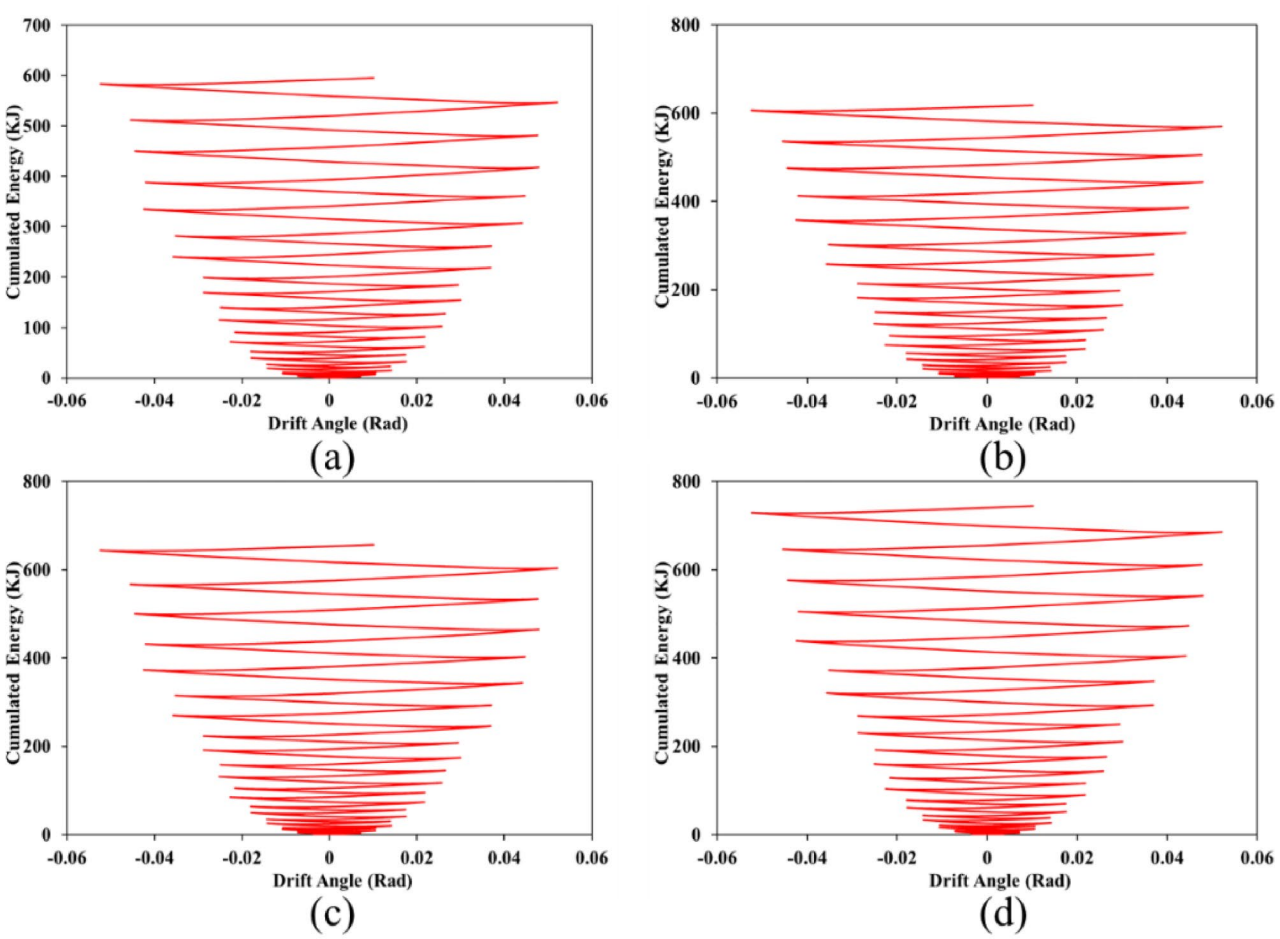
Cumulated energy of FCSSW configuration with the volume constraint of (**a**) 60%, (**b**) 70%, (**c**) 80%, and (**d**) 90%.

**Figure 32:**
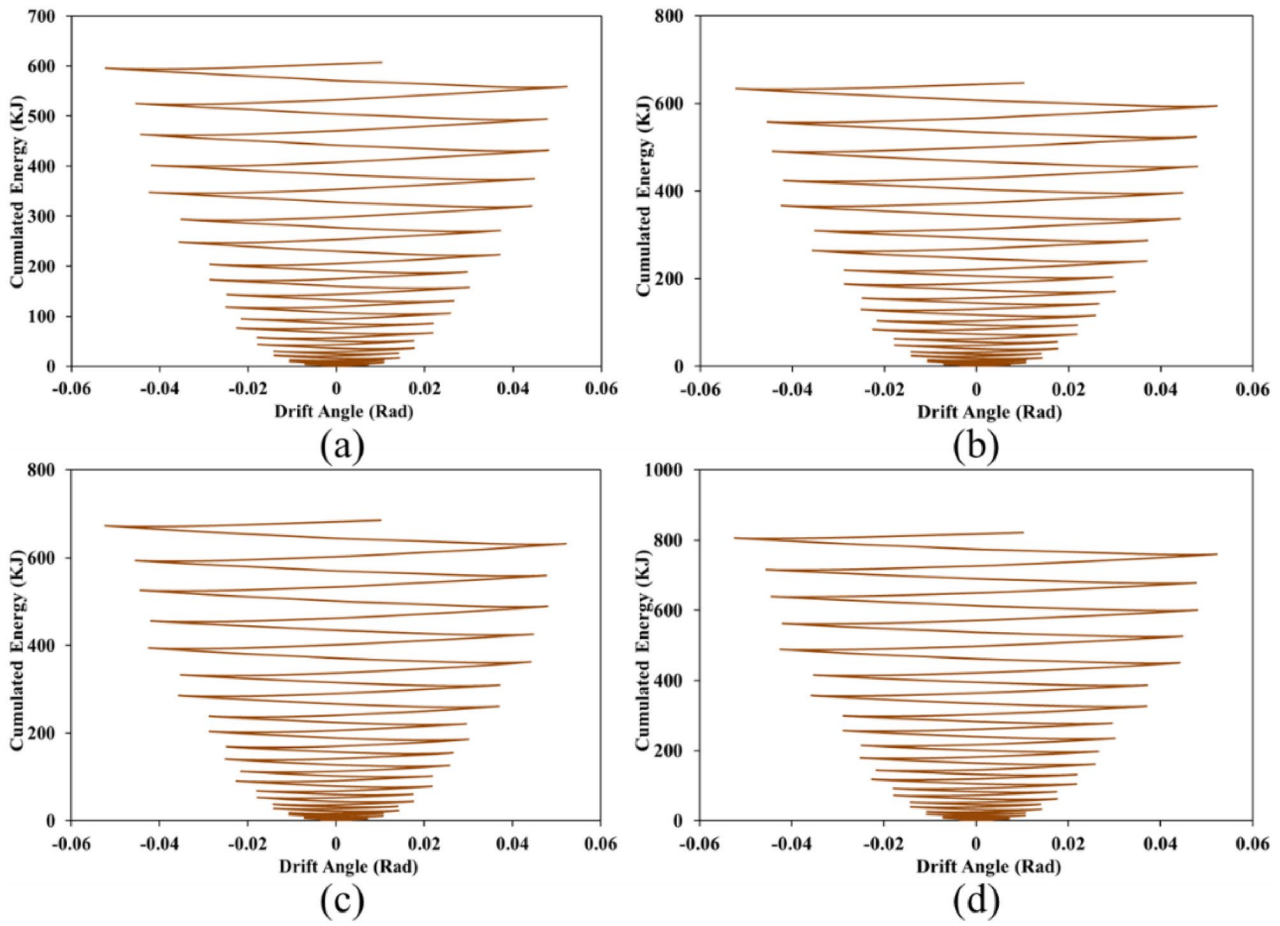
Cumulated energy of BCSSW configuration with the volume constraint of (**a**) 60%, (**b**) 70%, (**c**) 80%, and (**d**) 90%.

**Figure 33:**
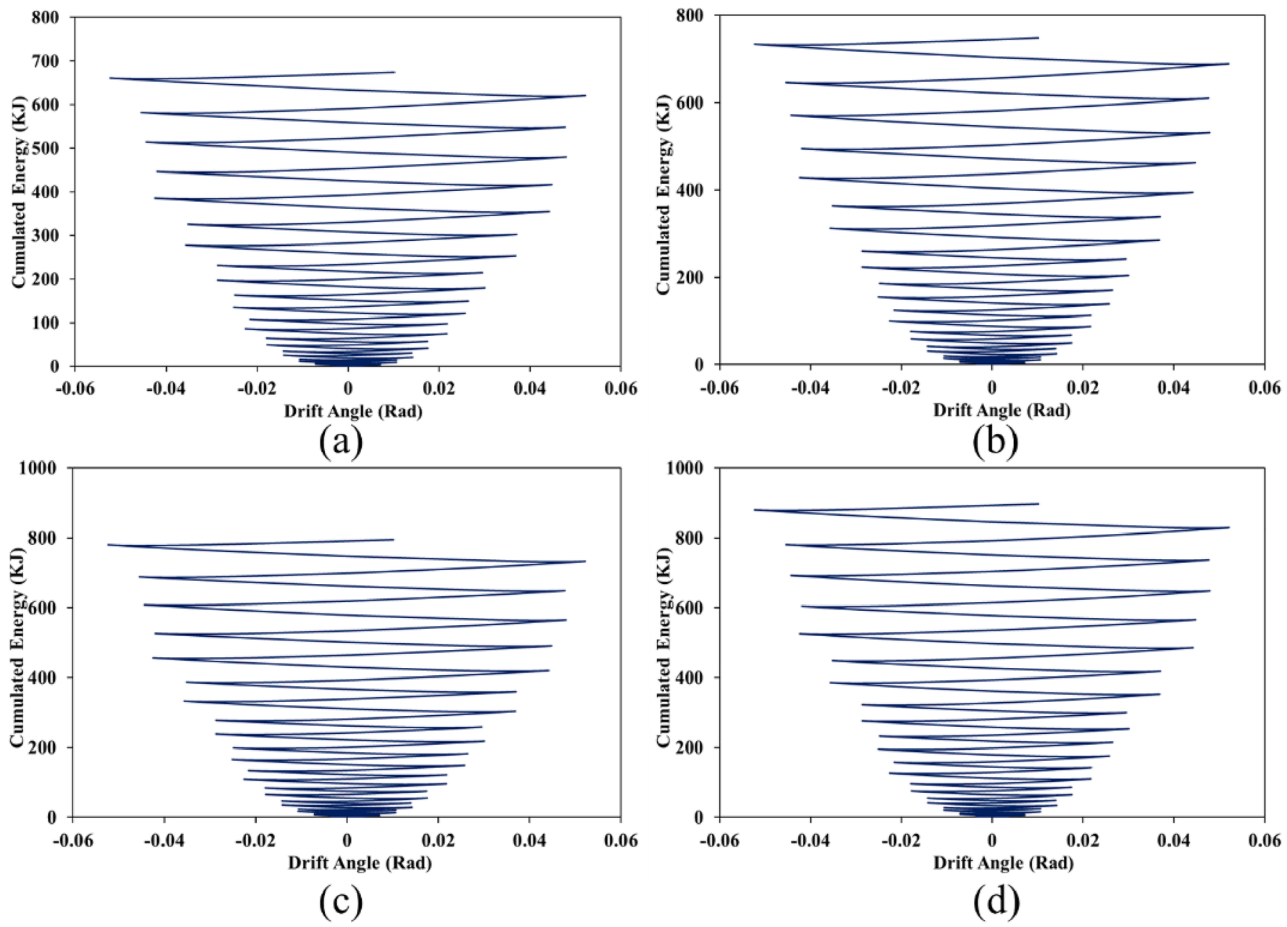
Cumulated energy of CCSSW configuration with the volume constraint of (**a**) 60%, (**b**) 70%, (**c**) 80%, and (**d**) 90%.

Each structure when exposes to the cyclic loading conditions, in each cycle, some exerted energy cumulates in the structure. As the structure absorbs more energy (i.e., cumulated one) means that the structure has a great performance against the seismic loads. Cumulated energy as a response to the SSWs was obtained from the FEA and presented in Figs. 31, 32, and 33 for configurations of FCSSW, BCSSW, and CCSSW, respectively.

Two important results were drawn from this subsection; first, the 90% volume constraint could result in higher amounts of cumulated energy, second, the CCSSW configuration was the best configuration among all three types of shear walls. Again, the frozen area in the CCSSW could resist the seismic loading sequences and absorb higher amounts of exerted energy.

For more detailed information, suppose a 60% volume constraint, all three types of TOs presented cumulated energy of around 600 kJ. Considering the FCSSW and BCSSW TOs resulted in cumulated energy of around 600 kJ but this value was increased up to 700 kJ for the CCSSW TOs. This issue was also repeated for other volume constraints. Thus, the CCSSW configuration presented good performance from a cumulated energy point of view. To conclude about obtained results, the plastic dissipation energies were calculated and plotted in the next subsection.

### Plastic dissipation energy

One of the most important aspects of SSWs is the high ductility during the loading conditions. The steel material presents ductile behavior due to its strain-hardening phenomenon. Thus, the SSWs show ductile behavior during earthquakes. It should be noted that any exerted load should be spent on a material and structure. There are two ways; absorbing exerted energy by producing the plastic zones and creating and expanding the cracks in a structure. Therefore, if a structure presents ductile behavior and consequently plastic deformation, the failure will be postponed.

**Figure 34:**
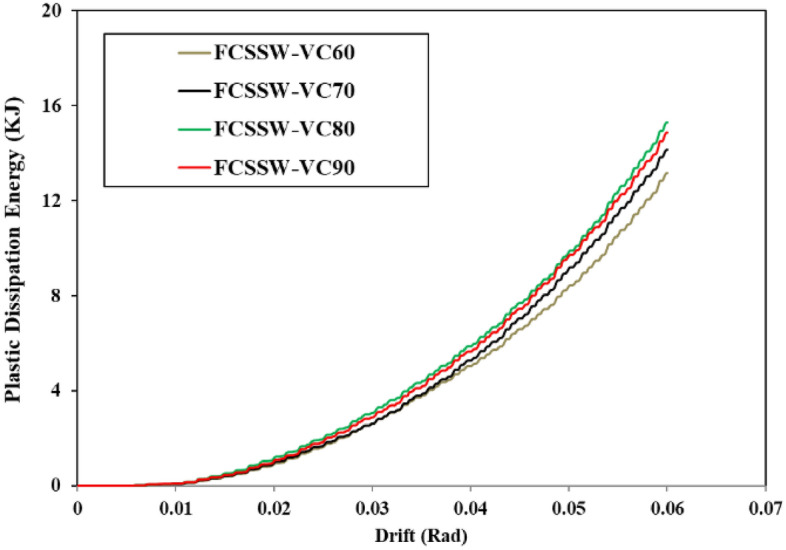
Plastic dissipation energy of FCSSW configuration with different volume constraints.

**Figure 35:**
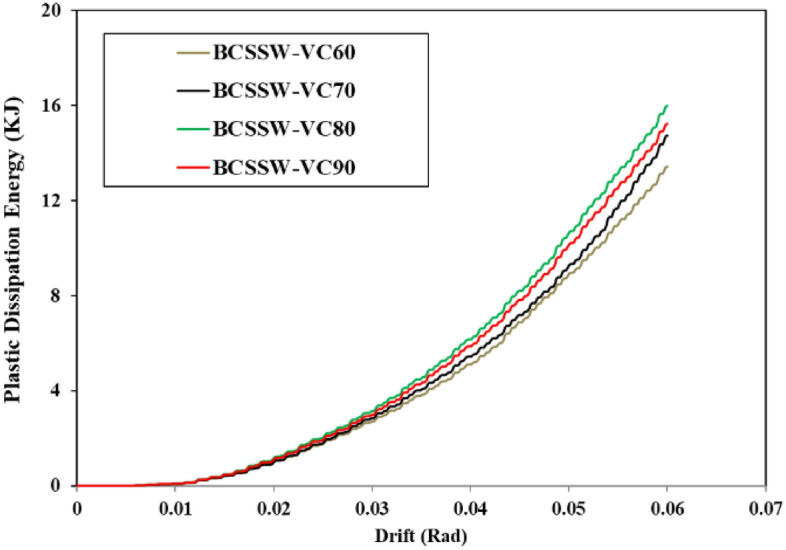
Plastic dissipation energy of BCSSW configuration with different volume constraints.

**Figure 36:**
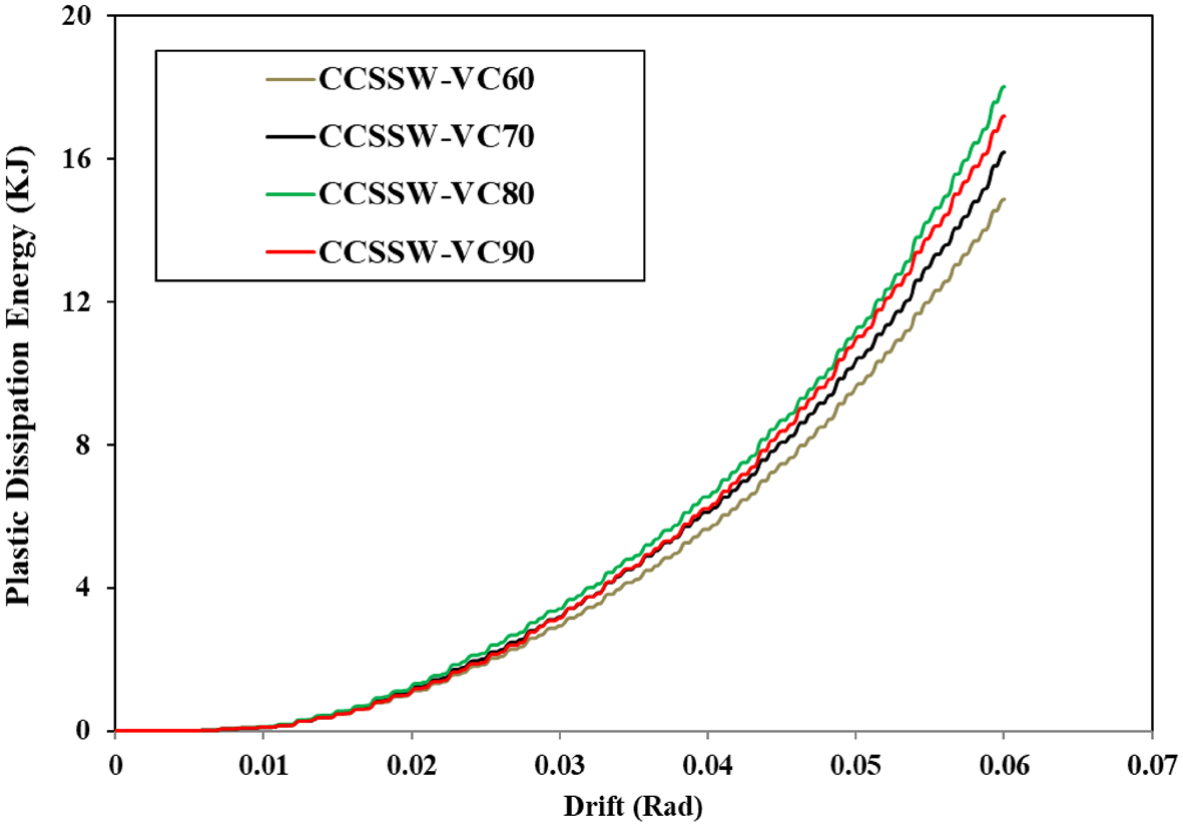
Plastic dissipation energy of CCSSW configuration with different volume constraints.

The plastic dissipation energy of different types of SSW was recorded from the FEA and plotted in the curves illustrated in Figs. 34, 35, and 36 for the FCSSW, BCSSW, and CCSSW configurations. It is obvious in these figures, that the 80% volume constraint resulted in higher plastic dissipation energy and then 90%, 70%, and 60%. The reason behind the lower plastic dissipation energy of the optimized SSWs with 60% volume constraint was due to the lower amounts of used material in this configuration. There was not enough steel material to absorb the exerted energy.

Another important result that was obtained here was the great performance of the CCSSW types from a plastic dissipation energy point of view. It is noteworthy that the main reason for these results was that the columns in this type were considered frozen in the TO procedure and the columns are the most important elements in the civil structures after the beams.

## Conclusions

The flat Steel Shear Walls (SSWs) are the common elements used in steel structures for damping the loads exerted by cyclic loading conditions like earthquakes. One of the important parts of the SSWs is the infill plate. In the current research paper, the Topology Optimization (TO) was used to optimize the shape of the steel infill plate of the SSWs. Two assumptions of strain energy and volume constraint were applied in the TO procedures for finding the good shape of the SSWs’ infill plates. Four volume constraints of 60%, 70%, 80%, and 90% were considered and besides, the strain energy was assumed as the maximized parameter during the seismic loading conditions. All these processes were done using the Finite Element Analysis (FEA) and the plugin named TOSCA. The main conclusions obtained in the current research paper are summarized here as below.

- The stresses were cumulated in the corners of the flat SSWs and conducting the TO could reduce the amounts of stresses in the concentration zones. Also, well dispersion of the stresses was observed for the optimized SSWs.
- The inside area of the optimized SSWs was less than the flat SSW. One of the discussed reasons for this issue was the lower weight of the optimized SSW compared to the simple one.
- Among the volume constraints, the 90% one could present higher cumulated energy and strength in a constant drift angle. However, 60% and 70% volume constraints could not present significant effects on the cumulated energy absorbed by the optimized SSWs.
- Plastic dissipation energy in the CCSSW was more than the other two TO configurations. Also, 80% volume constraint together with CCSSW configuration resulted in the highest value of plastic dissipation energy during the seismic loading conditions.

The results shown in the current research paper can be useful for the designers and engineers where there is a need for more efficiency with lower weight. For future studies, the experimental tests of the presented models in this paper can be considered. However, one of the challenges that the researchers may face is the fabrication of the optimized infill plates. It should be noted that the optimized infill plates can be prepared through the use of some manufacturing techniques such as laser cutting and sheet metal forming.

## Data Availability

All data generated or analysed during this study are included in this published article.
